# Children with Autism Spectrum Disorder and Abnormalities of Clinical EEG: A Qualitative Review

**DOI:** 10.3390/jcm13010279

**Published:** 2024-01-03

**Authors:** Chiara Bosetti, Luca Ferrini, Anna Rita Ferrari, Emanuele Bartolini, Sara Calderoni

**Affiliations:** 1 Department of Developmental Neuroscience, IRCCS Stella Maris Foundation, 56128 Pisa, Italy; chiara.bosetti@fsm.unipi.it (C.B.); l.ferrini5@studenti.unipi.it (L.F.); annarita.ferrari@fsm.unipi.it (A.R.F.); sara.calderoni@fsm.unipi.it (S.C.); 2 Department of Clinical and Experimental Medicine, University of Pisa, 56126 Pisa, Italy; 3 Department of Translational Research and of New Surgical and Medical Technologies, University of Pisa, 56126 Pisa, Italy; 4 Tuscany PhD Programme in Neurosciences, 50139 Florence, Italy

**Keywords:** EEG abnormalities, Autism Spectrum Disorder (ASD), neurodevelopmental disorders, children

## Abstract

Over the last decade, the comorbidity between Autism Spectrum Disorder (ASD) and epilepsy has been widely demonstrated, and many hypotheses regarding the common neurobiological bases of these disorders have been put forward. A variable, but significant, prevalence of abnormalities on electroencephalogram (EEG) has been documented in non-epileptic children with ASD; therefore, several scientific studies have recently tried to demonstrate the role of these abnormalities as a possible biomarker of altered neural connectivity in ASD individuals. This narrative review intends to summarize the main findings of the recent scientific literature regarding abnormalities detected with standard EEG in children/adolescents with idiopathic ASD. Research using three different databases (PubMed, Scopus and Google Scholar) was conducted, resulting in the selection of 10 original articles. Despite an important lack of studies on preschoolers and a deep heterogeneity in results, some authors speculated on a possible association between EEG abnormalities and ASD characteristics, in particular, the severity of symptoms. Although this correlation needs to be more strongly elucidated, these findings may encourage future studies aimed at demonstrating the role of electrical brain abnormalities as an early biomarker of neural circuit alterations in ASD, highlighting the potential diagnostic, prognostic and therapeutic value of EEG in this field.

## 1. Introduction

According to the criteria of the Diagnostic and Statistical Manual of Mental Disorders (5th Edition, Text Revision) [1], Autism Spectrum Disorder (ASD) is a neurodevelopmental disorder (NDD) with a prevalence of about 1% in the global population [2], characterized by persistent deficits in social communication and social interaction across multiple contexts and by the presence of restricted, repetitive patterns of behavior, interests, or activities. ASD symptoms must arise during the early period of development and cause clinically significant impairment in social, occupational, or other important areas of adaptive functioning. Furthermore, to make a diagnosis of ASD, these symptoms must not be explained by intellectual disability (ID), even if these two NDDs frequently co-occur: according to recent data from the Centers for Disease Control and Prevention, 37.9% of children with ASD also meet the criteria for ID [3]. In addition, ASD can frequently be associated with other NDDs (i.e., Attention-Deficit/Hyperactivity Disorder, language disorders, developmental coordination disorder, learning disorders) [4], or with a wide variety of neurological/somatic comorbidities (i.e., epilepsy, sleep problems, gastrointestinal disorders) [5]. In recent years, many genetic and environmental factors implicated in the pathogenesis of ASD have been identified [6,7], even if approximately 85% of individuals with ASD are still defined as idiopathic [8]. In this conceptual framework, an increasing number of scientific studies report a growing interest in characterizing neurobiological mechanisms possibly underlying ASD, including the alteration of neuronal proteins and brain circuits [9,10], in order to define useful biomarkers for early diagnosis and more effective treatment. For this purpose, many studies have recently focused on the use of neuroimaging techniques in infancy: firstly, to define canonical versus atypical developmental trajectories of the human brain, and secondly, to search for potential and valuable biomarkers of NDDs, as mentioned above [11]. Neurophysiological techniques can provide additional functional insights into ASD neurobiology. Amongst these methods, electroencephalogram (EEG) appears particularly attractive. It is a noninvasive tool, first introduced by Hans Berger in 1924 for human use, which allows for recording the electrical activity of the human brain derived from the summation of the excitatory and inhibitory postsynaptic potentials of neurons [12]. Datasets obtained with EEG can be assessed using visual examination and interpretation (qualitative EEG) or elaborated to obtain quantitative metrics (quantitative EEG). Both analysis techniques can be used in children with ASD, yet qualitative EEG is more directly linked to the possible occurrence of seizures [13,14].

As a matter of fact, the comorbidity of ASD with epilepsy has been extensively demonstrated over the last few years. The prevalence of epilepsy in people with ASD ranges from 1.8% to 60% [15,16], depending on several factors heterogeneously distributed within various study populations, such as:

- Type of EEG study: sleep EEGs are significantly more likely to detect epileptiform abnormalities than awake EEGs [17];
- Criteria used to make ASD diagnosis: the prevalence of epilepsy in people with a diagnosis of autism based on Kanner’s Autism criteria is likely to be much higher than the prevalence of epilepsy in individuals with a diagnosis of ASD based on DSM-5 criteria [18] since severe Autism Spectrum Disorder symptoms represent an independent risk factor for epilepsy [19];
- Co-occurrence of ID: not only is epilepsy more frequent in ASD patients with ID, with a prevalence three times greater in people who have both ASD and ID than in people who have ASD but not ID [20], but its rate also increases as IQ decreases, with the highest rate when IQ < 40 [21,22];
- Developmental regression: despite the fact that the relationship between regression and epilepsy in autism remains unclear, several studies report statistically significant associations between the presence of regression and an increase in epilepsy [23];
- Age: despite the classic bimodal distribution of epilepsy according to age (i.e., early childhood and adolescence), a significantly higher prevalence of seizures in ASD is detectable during adolescence [24]. Accordingly, a large study reported that the overall prevalence of epilepsy in ASD children aged 2 to 17 is 12.5%, but this rate is largely driven by epilepsy in ASD children aged 13 to 17, in which the prevalence is 26% [25];
- Gender: the risk for epilepsy appears to be significantly higher for females [26]; while the overall male-to-female (M:F) ratio frequently reported is approximately 4:1 [2,3,27], the M:F prevalence ratio decreases to 2.5:1 in samples of individuals with both ASD and epilepsy [28];
- Idiopathic versus non-idiopathic ASD: the prevalence of epilepsy in syndromic autism is typically higher than in idiopathic autism [23].

In the last decade, the co-occurrence of ASD and epilepsy has also pioneered the demonstration of the common neurobiological bases that these disorders seem to share.

It has been widely demonstrated that an imbalance between excitatory and inhibitory neurotransmission can be found in people with epilepsy, involving glutamate (Glu, excitatory circuits) and gamma-aminobutyric acid (GABA, inhibitory circuits) as the main neurotransmitters implicated in the epileptogenesis through many different mechanisms of alteration [29,30,31,32,33,34,35,36,37], but recently, some studies also focused on other neurotransmitters (i.e., acetylcholine) and glial cells, in an attempt to better explain the neurobiological basis of epilepsy [34].

Interestingly, there is some convincing evidence that most of the aforementioned alterations can be also found in the brain tissues of ASD patients [31,38,39,40,41,42].

Moreover, most of the neurobiological bases shared by ASD (and, more broadly, neurodevelopmental disorders) and epilepsy likely originate from common genetic causes, which can explain the altered expression of a large variety of proteins involved in neurotransmission. For example, variants of the gene *GABARD* (encoding for the delta subunit of GABA-A receptors) would predispose to both ASD and generalized epilepsy [43]. Other genes involved in the early stages of brain development and migration of neuronal progenitors have been associated with both ASD and epilepsy (i.e., the *CYFIP1* gene, the CHD5 gene, CASPR2 and other genes coding for neurexins) [44,45,46,47].

According to the scientific literature, the presence of epileptiform—and sometimes also non-epileptiform—abnormalities on the first EEG performed seems to be predictive of an increased risk of subsequent and earlier onset epilepsy. This is self-explicating in conditions [48,49,50] characterized by a predisposition to epilepsy per se co-occurring with ASD symptoms, such as the Tuberous Sclerosis Complex (TSC). In TSC, EEG abnormalities occurring early during the disease course predict the development of epilepsy.

Conversely, it is difficult to demonstrate that early EEG abnormalities predict unprovoked seizures in children with idiopathic ASD. Indeed, EEG could be interpreted as a biomarker of epileptogenesis, considering epileptiform EEG discharges may predate epilepsy in children with febrile seizures [51,52] and are associated with further seizures in children with a first clinical episode [53]. Theoretically, EEG could also be related to the neurodevelopmental outcome, even though there is still an important lack of evidence in this field and further studies to confirm its role as a predictive biomarker in neurodevelopmental disorders are needed.

A prevalence of 8–80% of abnormalities on EEG, such as Isolated Epileptiform Discharges or Paroxysmal Slowing Activity, has been documented in non-epileptic children with ASD [14,54]. In recent years, several studies have tried to demonstrate the role of these electric brain abnormalities as a possible biomarker of altered neural connectivity in people with ASD. Based on this background, the main purpose of the current narrative review was to examine and summarize the recent scientific literature concerning EEG findings in children/adolescents with ASD. In particular, we focused on studies about standard EEG data obtained from cohorts of patients with idiopathic ASD.

## 2. Materials and Methods

### 2.1. Literature Research

We carried out research using three different databases (PubMed, Scopus and Google Scholar), following, although not faithfully, since this is a qualitative review, the Preferred Reporting Items for Systematic Reviews and Meta-Analysis (PRISMA) guidelines. We restricted the publication period from 2013 to 2023; the latest database research was performed in June 2023. As search terms, we used EEG OR Electroencephalography AND Abnormalities AND Children AND Autism OR ASD.

We identified 696 records (306 in Pubmed, 299 in Scopus and 91 in Google Scholar). After a first skimming, excluding 586 articles based on the title and removing all duplicates from the remaining 110 records, we obtained 44 articles. These articles were then screened manually and independently by two reviewers and assessed for eligibility according to the pre-established criteria outlined below.

### 2.2. Inclusion Criteria

- ASD diagnosis based on DSM criteria or ICD criteria.
- Cohort including participants with idiopathic ASD.
- Participants with ASD as the main diagnosis.
- Articles published in the English language.
- Availability of a full text of the paper.

### 2.3. Exclusion Criteria

- Studies focused on quantitative EEG (qEEG).
- Non-ASD participants.
- The age range of ASD patients having an upper limit greater than 18 y or the age range not specified.
- Meta-analyses or literature reviews.

## 3. Results

**Figure 1:**
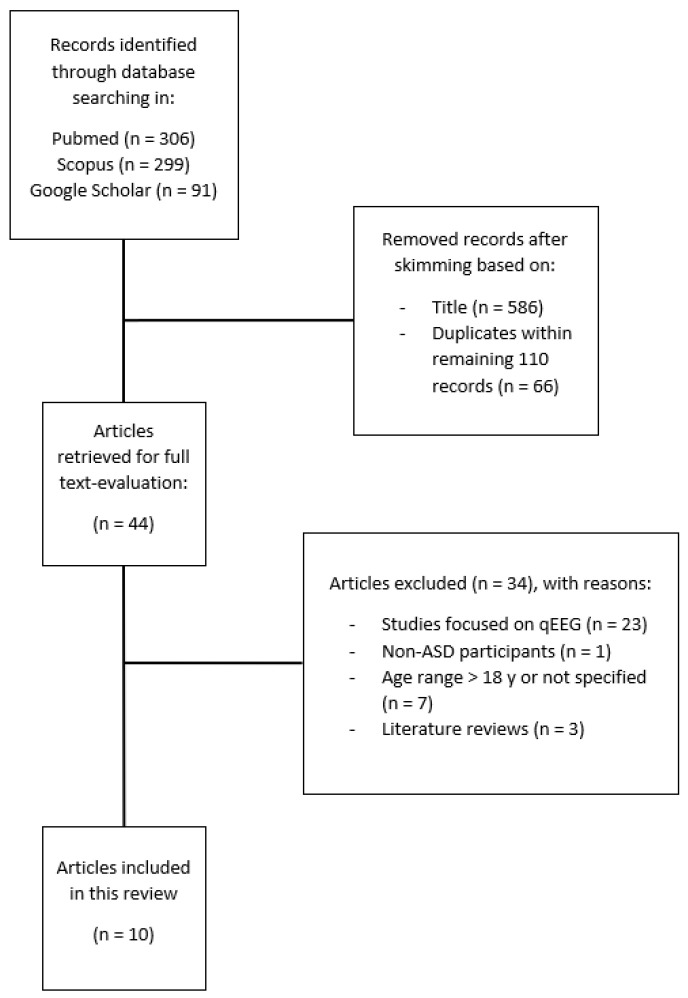
Flowchart of the article selection process.

After a full-text evaluation, 10 original articles [52,54,55,56,57,58,59,60,61,62] were included in this qualitative review, as reported in detail in (Figure 1).

Table 1 summarizes the most relevant demographic and clinical information of the included articles.

### 3.1. Characteristics of Participants

ASD diagnoses were made according to the DSM-IV or DSM-IV-TR criteria [58,61,63], DSM-5 criteria [54,55,56,57,59,62] or ICD-10 criteria [60]; eventually, in some cases, additional well-established assessments for autism were performed to corroborate the diagnosis, such as the administration of the Autism Diagnostic Observation Schedule—Second Edition (ADOS-2) [54], Autism Diagnostic Interview—Revised (ADI-R) [54,60] or the Childhood Autism Rating Scale (CARS) [58].

Regarding the age range of participants, only two studies focused on children aged 5 years or less [54,57].

Four studies included more than 100 ASD participants [54,56,60,62]. The remaining six studies considered a cohort ranging from 21 to 90 autistic patients. Both females and males were included in all studies, despite a constant imbalance between the sample size of these two populations, with males always outnumbering females.

**Figure 2:**
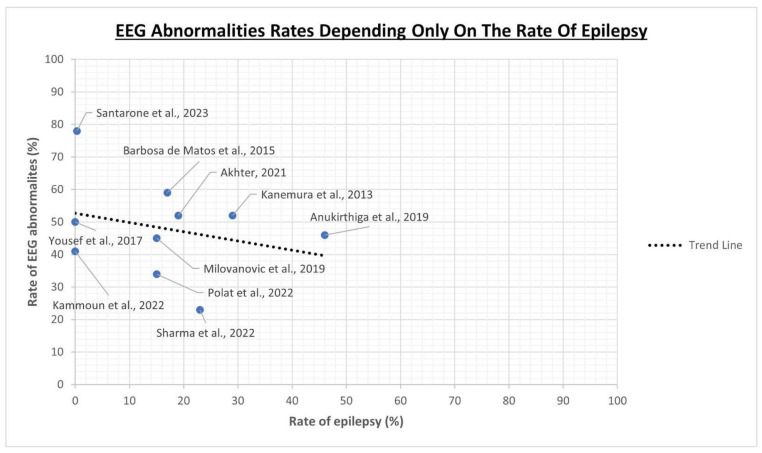
Relationship between EEG abnormality rates and only the rate of epilepsy as a variable taken independently of the rest [54,55,56,57,58,59,60,61,62,63].

The rates of epilepsy in the study cohorts we selected were quite variable, ranging from 0.3% to 46%, and two studies [55,58] did not include patients with epilepsy. Despite the fact that we might expect a higher rate of EEG anomalies in those cohorts with a higher number of epileptic patients, the data examined did not show this type of relationship. As it can be seen in Figure 2, by relating the rate of EEG abnormalities only with the rate of epilepsy, we could be mistakenly led to think, paradoxically, that a higher rate of epilepsy is associated with a lower rate of EEG abnormalities. This is due to the fact that, as we will more thoroughly discuss later in this manuscript, the rate of EEG abnormalities, as well as the rate of epilepsy itself, are also influenced by other variables heterogeneously distributed in the populations under study, such as the severity of the autistic phenotype, the functional profile and the presence of other neuropsychiatric comorbidities. This makes it complex to understand the role of epilepsy in the interpretation of the results.

### 3.2. EEG Technical Issues

The majority of papers included in our review mention the use of digital EEG to carry out recordings of brain electrical activity in patients, with the most widespread International 10–20 system electrode placement on the scalp. However, we observed extreme variability in the specific EEG equipment used in various studies, even if this issue is not frequently specified.

Regarding the recording condition, in three studies, the patients only underwent sleep recordings [55,58,61]; three studies considered the sleep/wakefulness condition [54,56,62], where one of them also performed only wakefulness recording [62]; two studies could not ensure sleep and wakefulness conditions in all participants [59,60]; and two remaining papers did not specify the baseline condition [57,63]. In some of the studies mentioned above, sleep was pharmacologically induced, at least for poorly cooperative children [58,59,60]. In addition, sleep deprivation before the exam was only mentioned by Kammoun et al., 2022 and Anukithiga et al., 2019. Other activation procedures were mentioned in three studies and consisted of hyperventilation and photic stimulation [56,59,60].

The duration of recording, when specified, ranged from a minimum of 20 min [60] to a maximum of 70 min [54].

### 3.3. Main EEG Findings

All the studies highlighted the presence of epileptiform discharges with heterogeneous figures. Seven studies also found non-epileptiform abnormalities [54,55,56,57,60,61,62]. Only two studies [54,60] specified if EEG abnormalities were found during wakefulness, during sleep or both.

In particular, every study found that Inter-Ictal Epileptiform Discharges (IEDs) were heterogeneously located in almost all cortical areas. However, five out of ten studies explicitly reported the presence of multifocal abnormalities [55,57,59,62,63], and eight studies reported the presence of generalized discharges [54,56,57,58,59,60,61,62]. The type of epileptiform discharges seems to be deeply heterogenous, varying from slow waves [54,56,58,61] and spike waves [54,56,58,60] to spikes, polyspikes and polyspike waves [55,56,60,61,62] and also to sharp waves [56,58,61,62].

Regarding non-epileptiform abnormalities, Barbosa de Matos et al., 2015 [57] highlighted the presence of mild–moderately disorganized background activity in two patients of their cohort. Similarly, Milovanovic et al., 2019 [60] found an abnormal background activity in 14 patients and a focal slowing of electrical activity in one subject, both during wakefulness. Moreover, they also described an altered sleep architecture in four patients of their cohort. Sleep disorganization was also observed by Kammoun et al., 2022 [55], consisting of the asynchrony of sleep spindles or, in general, a poorly organized electroencephalogram.

In his study, Akhter detected non-epileptiform abnormalities on EEG recordings in eight subjects (15.4% of total subjects), described as a slowing of background theta/delta waves, generalized intermittent slow waves, excessive beta activity and lateralized asymmetry. Polat et al., 2022 and Sharma et al., 2022 both highlighted abnormal delta activity, consisting of a paradoxical delta rhythm [56] and/or a slowing of the same activity [62]. Santarone et al., 2023 [54] described non-epileptiform abnormalities both during wakefulness (slow or irregular background activity, asymmetry, abnormal fast activity) and during sleep (slow or irregular background activity, asymmetry, asynchrony, abnormal fast activity).

With respect to EEG abnormalities’ localization, we observed a wide variability in cortical sites where such anomalies were detected: frontal regions were mentioned in all the eight studies considered in this review that specified the localization of EEG findings [54,55,56,57,58,61,62,63], variously accompanied by central, frontocentral, frontotemporal and frontoparietal abnormalities [54,56,58,61,62]. In addition, temporal [54,55,57,62], centrotemporal [58,61,63] and centro-temporoparietal [61] were involved, as well as centro-parietal [58,61] and occipital [54,55,61,62] areas.

### 3.4. Correlation between EEG Abnormalities and Clinical Features

Some papers also investigated the possible association between EEG abnormalities and noteworthy clinical features pertinent to the neuropsychiatric field. Regarding the possible correlation between types of EEG abnormalities and ASD severity, only Yousef et al., 2017 [58] put forward the hypothesis of a linkage between these features: in particular, in severe ASD, generalized abnormalities seemed to be predominant, followed by bilateral frontotemporal and bilateral centrotemporal alterations. Furthermore, Kammoun et al., 2022 [55] maintained that, in their cohort of patients, all those who showed EEG abnormalities had been diagnosed with moderate–severe ASD, thus vaguely speculating on a possible association between ASD severity and the presence of abnormalities on an EEG recording. Taken together, the small amount of information about the main field that we aimed to detect in this narrative review cannot be generalized and thus emphasizes a lack of data in the literature regarding a possible significant association between EEG abnormalities and the phenotype of idiopathic ASD in children/adolescent populations.

Regarding other neuro-developmental comorbidities, Anukirthiga et al., 2019 [59] showed a significant association between IEDs and the intellectual quotient (IQ); in more detail, IEDs and epilepsy were significantly more common in subjects with an IQ below 80. According to Akhter, ASD patients with epileptiform or non-epileptiform abnormalities showed a more frequent association with intellectual disability (ID), especially moderate or severe ID. Kammoun et al., 2022 [55] reported an association between EEG abnormalities and behavioral disorders, the absence of language or language regression and ID in ASD patients who took part in their study. In particular, 81.81% of patients with behavioral disorders showed EEG abnormalities located in the frontal lobe; subjects with an absence of language showed IEDs in different lobes and sleep disorganization seemed to correlate with language regression and behavioral problems.

According to Santarone et al., 2023 [54] there is a non-significant association between developmental delay and IEDs; on the other hand, they showed a statistically significant correlation between abnormal background activity during sleep and developmental delay in ASD patients.

Only two of the studies were longitudinally designed and followed-up patients for at least six months. New EEG recordings confirmed the data collected during the first experimental session or, in some cases, highlighted some modifications, variably consisting of a worsening or an amelioration in EEG features, which were sometimes related to epilepsy development after the first recording [56,63].

## 4. Discussion

The results shown in Table 1 and summarized in the previous paragraph turned out to be very heterogeneous and not systematically comparable to each other.

### 4.1. Effect of ASD Diagnostic Criteria on the EEG Abnormality Rate

The first thing that stands out is the presence of a certain heterogeneity regarding the diagnostic criteria used to identify patients with autism: this aspect significantly influences the percentage of EEG abnormalities found in the various study cohorts. The rate of EEG abnormalities correlates, indeed, with the severity of ASD [18,55,58]: the greater the number of patients with severe ASD, the higher the rate of EEG abnormalities will tend to be. To understand what the problem is, it is necessary to remember that DSM-IV [64], DSM-IV-TR [65] and ICD-10 [66] referred to autism as a member of the Pervasive Developmental Disorders group, which included different nosographic entities characterized by different severity. In DSM-5 [67], this academic subdivision was abandoned, and these disorders, which actually represent the spectrum of the fundamental *core* symptoms of autism, were grouped under the single name of Autism Spectrum Disorder.

For patient selection, two articles included in this review referred to DSM-IV [58,63], one to DSM-IV-TR [61] and one to ICD-10 [60]: the first three aforementioned studies only included patients with Autistic Disorder, while the last one only included individuals with Childhood Autism. These two names refer to the same disease, which can also be referred to as Kanner’s Autism: this definition refers to the most severe type of autism, which, not surprisingly, is associated with a greater prevalence of epilepsy [18] and EEG abnormalities. The remaining six articles [54,55,56,57,59,62], instead, referred to DSM-5 criteria; therefore, we found patients with a much more heterogeneous degree of severity of the disease. For example, it was possible to find in the same cohort both low-functioning and high-functioning ASD patients (that, in the past, would have fallen under two different diagnoses—Autistic Disorder and Asperger Disorder, respectively) in proportion to each other, often not even made explicit by the authors.

**Figure 3:**
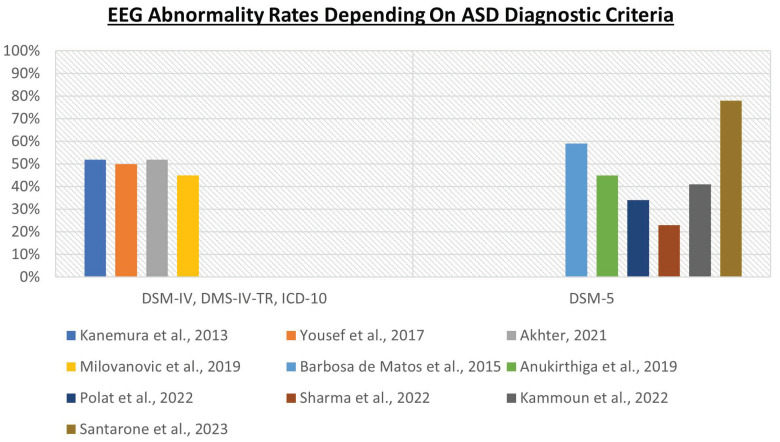
The criteria used to diagnose ASD influence the EEG abnormality rates [54,55,56,57,58,59,60,61,62,63].

This substantial difference between these two groups of studies is reflected in a different distribution of the rates of EEG abnormalities, as graphically represented in Figure 3. Within the cohorts selected based on DSM-IV, DSM-IV-TR and ICD-10 criteria, and with more homogeneous phenotypic characteristics, the rates of EEG abnormalities are more consistent, with a range from 45% [60] to 52% [61,63] and an average of 50%. On the contrary, within the cohorts selected based on DSM-5 criteria, in which patients have very heterogeneous phenotypic characteristics, the rates of EEG abnormalities are extremely variable, with a range from 23% [62] to 78% [54] and an average of 47%.

### 4.2. Neurophysiopathological Basis of the Link between ASD and EEG Discharges

The link that unites the severity of the autism phenotype and EEG abnormalities has its roots in the pathophysiological consequences of epileptic discharges. Jarero-Basulto et al., 2018 [68] carried out a literature review that analyzes the close relationship between epilepsy and neuroplasticity. In samples affected by Temporal Lobe Epilepsy (TLE), obtained from animal models and human post-mortem brains or post-operative specimens [69], several authors found the presence of anomalous neuronal circuits in the hippocampal region. It seems that epileptic discharges, not necessarily long-lasting (as in the case of Status Epilepticus) but recurring over time, are capable of determining neuronal death and axonal sprouting in the affected area. The latter, according to some authors, is a reaction to neuronal death [70] but, according to others, the discharges themselves, without the involvement of neuronal death, can trigger it [71]. Neuronal loss and/or the formation of new synapses will lead to the development of aberrant neuronal circuits, characterized by an excitation/inhibition imbalance. This alteration could be due to various mechanisms:

- More marked reduction in inhibitory GABAergic interneurons, which would be more susceptible to death induced by epileptic discharge than excitatory glutamatergic neurons [68];
- Reduction in inhibitory GABAergic transmission: along with the concomitant reduction in the levels of GABA-A receptor ligands and the activity of glutamate decarboxylase (GAD) [31], this phenomenon is also the result of glutamate accumulation, due to the hyper-synchronism of epileptic discharge that causes glutamatergic hyperstimulation of post-synaptic neurons. These neurons undergo an increase in intracellular [Ca^2+^] and the consequent activation of Calcineurin which, through dephosphorylation, causes the internalization of post-synaptic GABA-A receptors [72];
- Deregulation/alterations of the structure of glutamate receptors [35,36,37];
- Lack of an excitatory-to-inhibitory shift in GABA during early brain development after birth [31,32];
- Reduction in Parvalbumin (PV) levels: this event may be linked to the reduction in the number of Parvalbumin-expressing (PV+) GABAergic interneurons, which according to some authors, would be more susceptible to death from excitotoxicity [73], or linked to the reduction in the mRNA coding for PV in the absence of a real reduction in the number of PV+ GABAergic interneurons [74].

This excitation/inhibition imbalance, on the one hand, would, in turn, facilitate the appearance of new epileptic discharges, and, on the other hand, would predispose individuals to the development of other psychiatric comorbidities, including ASD [75,76,77], Major Depressive Disorder, anxiety and psychosis [78].

One of the most suggested neurobiological mechanisms of ASD pathophysiology consisted of an imbalance between excitation and inhibition signaling, of which the nature is still a subject of debate. The first hypothesis, formulated by Rubenstein and Merzenich in 2003 [75], supports the prevalence of the excitatory component, but other authors subsequently observed the prevalence of the inhibitory component [76], at least in some particular types of autism, such as the one linked to Rett Syndrome.

From this point of view, a very interesting role is played by Parvalbumin (PV), which is a Ca^2+^-binding protein belonging to the EF-hand superfamily: it is mainly located in the cytoplasm, but extracellular isoforms also exist [79]. Parvalbumin can be found in many different cells, including type-II muscle fibers, kidney cells, some cells belonging to the endocrine system, myocardiocytes, cells of the inner ear and some neurons of the Peripheral Nervous System (PNS) and Central Nervous System (CNS). The latter includes Parvalbumin-expressing (PV+) GABAergic interneurons, which represent the largest class of inhibitory GABAergic neurons in the CNS: they are fast-spiking cells that, in the cerebral cortex, provide feedforward and feedback synaptic inhibition onto a diverse set of cell types, including pyramidal cells, other inhibitory interneurons and themselves [80]. More precisely, some of these Parvalbumin-expressing (PV+) GABAergic interneurons, represented by PV+ Chandelier Cells and PV+ Basket Cells, appear to have the function of synchronizing the activity of various cortical pyramidal cells [81] through their rhythmic inhibition.

Abnormalities affecting Parvalbumin-expressing (PV+) GABAergic interneurons cause an excitation/inhibition imbalance, which correlates with the autistic phenotype [82,83]. In particular, two diametrically opposite effects can occur [83]: the loss of PV+ GABAergic interneurons determine an imbalance in favor of excitation, while the reduction in PV expression levels, in the absence of an effective reduction in the number of interneurons, is responsible for an imbalance in favor of inhibition. Therefore, these abnormalities could explain both hypotheses relating to the excitation/inhibition imbalance [75,76] and the debate today is still open. For example, Hashemi et al., 2016 [84] found a significant reduction in the number of PV+ GABAergic interneurons in some cortical areas of ASD patients, while Filice et al., 2016 [83] argued that the reduction in PV expression levels, in the absence of an effective reduction in the number of PV+ GABAergic interneurons could represent an element common to some forms of ASD.

Interestingly, in animal models (rats), it has been observed that, by inducing epileptic seizures with 4-aminopyridine administration, it is possible to reduce the expression of PV [74]. These data seem to strengthen the hypothesis of a pathophysiological link between EEG abnormalities and atypical neurodevelopment in ASD subjects.

### 4.3. Relationship between EEG Abnormalities and the ASD Phenotype

The hypothesis stating that epileptic discharges have the ability to modify neuronal circuits has found wide support in the literature. Therefore, given the notable prevalence of epilepsy and EEG abnormalities in the ASD population, it is important to investigate the possible role that EEG abnormalities could play in the pathophysiology of autism. Indeed, several studies have addressed the possible relationship between EEG and the autism phenotype.

EEG abnormalities can be divided into ictal abnormalities, when their occurrence is associated with seizures, and interictal abnormalities. The latter can in turn be distinguished into epileptiform and non-epileptiform. This heterogeneity also recurs within the ten articles we selected: four studies do not specify which particular anomalous graphoelements were found [54,57,59,63], while the remaining six studies provide rather inconsistent classifications. This is disadvantageous from the perspective of researching a potential EEG biomarker of autism since, in order to identify a possible association between ASD and EEG abnormalities, it should be clarified which type of abnormalities were found in the various studies so that they can be classified in the exact same way, so as to reduce the subjectivity of interpretation. Similarly, it would be very important to specify the location of the EEG anomalies, as different locations can be associated with different phenotypic aspects. In ASD patients, EEG abnormalities have been found in all four brain lobes, which are involved in carrying out different functions [85,86,87,88,89].

The impairment of both the temporal and frontal lobe may drive the core symptoms of ASD such as the alteration in social functions and ability to process emotions and facial expressions, nonverbal communicative behaviors and relational skills and executive functions [90,91]. However, the underlying pathophysiology may derive from an aberrant connectivity between different brain regions rather than a straight morphological alteration of brain structures [92]. The comorbidity between ASD and epilepsy may also be influenced by aberrant connectivity between different brain regions. People with Frontal Lobe Epilepsy (FLE) and Temporal Lobe Epilepsy (TLE) may also exhibit specific neurodevelopmental features partially overlapping with the ASD spectrum (e.g., behavioral disorders, attention liability, alteration of executive functions, intellectual disability, language impairment or memory impairment) independently from seizure occurrence. In particular, people with FLE may be particularly prone to deficient executive functions and memory impairment, suggesting the involvement of an underlying neuronal circuitry of the frontal lobe. FLE patients may present anomalies that mainly concern visuospatial organization, planning ability, response inhibition, impulse control, working memory, verbal search, mental flexibility and programming of complex motor sequences. All of this can also lead to the development of difficulties in mathematical calculation and reading [93]. Regarding language, some authors [94] maintain that FLE patients present an initial temporary impairment of linguistic understanding associated with persistent impairment of linguistic production, while others [95,96,97] believe that impaired verbal search and impaired verbal fluency are also associated. Regarding memory impairment, some authors [98] maintain that long-term epileptic activity constitutes a risk factor for this anomaly. Regarding intellectual disability, some authors [97,99,100,101] maintain that FLE is associated with a reduction in IQ, while others [102] believe that IQ is not compromised. What is most interesting is the fact that these anomalies, when present, tend to undergo remission following anti-epileptic treatment [95]: this suggests that they are closely linked to epilepsy. It has been observed that in the ASD population, epilepsy correlates with behavioral disorder severity [103], as well as, significantly, with the phenomenon of autistic regression [17].

As mentioned for behavioral problems, cognitive impairment, in all its facets, can also be found in ASD patients [67,104,105,106,107,108,109,110,111,112,113,114,115,116,117,118,119,120,121]. In ASD patients, ID is significantly associated with epilepsy [122,123], and the prevalence of the latter is higher in ASD patients with ID compared with ASD patients without ID [26]. In addition, the presence of epilepsy is a significant factor in ID severity [15,17,124,125,126,127,128]. In ASD patients, epileptic discharges often affect the frontal lobe [129], causing a potential frontal lobe dysfunction which, as previously mentioned, could explain some traits of the autistic phenotype [130,131].

Given what has been said so far, it is reasonable to assume that epileptic discharges can interfere with brain maturation during childhood, shaping a child’s phenotype even up to the onset of pathological conditions. A reduction in PV+ GABAergic interneurons [84] has been found in the pre-frontal cortex of ASD subjects, which, as we already mentioned, can be triggered by epileptic discharges repeated over time [74]. This aspect, in addition to predisposing a patient to an excitation/inhibition imbalance [84], typical of ASD, is also associated with a greater incidence of anxiety-like behaviors [132] in animal models, which are part of the typical comorbidities of ASD patients [78].

Leaving epilepsy aside, it is important not to neglect SEAs, which are present in both epileptic and non-epileptic ASD patients; among the latter, they show a prevalence varying from 8% to 60.7% [17,60,109,126,133,134,135,136,137,138,139,140,141,142,143,144,145]. This variability is probably due to sampling and methodological heterogeneity in collecting and interpreting EEG tracings [23]. Although these abnormalities can also be found in healthy individuals [146,147,148], they are significantly more frequent in ASD patients, who exhibit them in all four cerebral lobes. According to some authors [141], the most frequent site is represented by the temporal lobe, but another study [149] reports that the first position is occupied by the frontal lobe with a rate of 78%.

What was just stated can also be deduced from the articles selected in this review, as all, except for one [57], report the presence of non-epileptic ASD patients with SEA in their EEG tracings. Two of these studies [59,60] do not specify the location of the abnormalities, but in all of the others, the most common ones are represented by temporal, frontal and frontotemporal areas, with an order of frequency varying between the different studies. Some of them also highlight a certain recurrence between epileptiform SEAs and some phenotypic characteristics, which follows what we already mentioned for epilepsy:

- Yousef et al., 2017 [58] argue that generalized EEG abnormalities are the most frequent, followed by focal ones: in severe ASD, they are typically bilateral fronto-temporal or centro-temporalI; in moderate ASD, they are typically frontotemporal and centroparietal; and in mild ASD, they are typically centroparietal;
- Anukirthiga et al., 2019 [59] maintain that epileptiform SEAs, as well as epilepsy, are significantly more frequent in ASD patients with an IQ less than 80;
- Milovanovic et al., 2019 [60] assert that epileptiform SEAs, like epilepsy, have small effects on motor skills and no effect on adaptive behavior or communication/socialization/daily living skills;
- Akhter, 2021 [61] reports that epileptiform SEAs can be found in ASD patients both with ID and without ID, but they tend to be more frequent in subjects suffering from moderate–severe ID;
- Kammoun et al., 2022 [55] report that all patients with EEG abnormalities suffered from moderate–severe ASD and that, more specifically, ten subjects showed behavioral instability, which was associated with EEG abnormalities in the frontal lobe in 81.81% of them; twelve subjects showed absence of language, which was associated with EEG abnormalities in different lobes; four subjects showed language regression; and nine subjects showed ID;
- Santarone et al., 2023 [54] claim that there is no significant association between SEAs and developmental delay in ASD patients.

Regarding non-epileptiform SEAs, some authors [150] assert that they are associated with a less severe phenotype compared with epileptiform SEAs: ASD patients with epileptiform SEAs perform worse on executive functioning assessments and exhibit higher scores in inhibition self-control compared with the ones reporting non-epileptiform SEAs. Among the articles we selected, only two of them relate non-epileptiform SEAs to the phenotype: Akhter, 2021 [61] reports that they can be found in ASD patients both with ID and without ID and Santarone et al., 2023 [54] argue that there is a significant association between abnormal background activity during sleep and developmental delay. This follows a widespread trend in the scientific literature, which focuses above all on the role of epileptiform abnormalities, attributing less importance to non-epileptiform ones. The number of studies that focus on the latter, in fact, is small compared with the impressive number of articles focusing on the former, which report contrasting opinions between them.

Santarone et al., 2023 [54] argue that in ASD patients, epileptiform SEAs should be considered a nonspecific sign of cortical dysfunction, even in the absence of clinical epilepsy, but their actual role is still subject to debate. As a model, Numis et al., 2011 [151] considered patients affected by Tuberous Sclerosis Complex (TSC), which is an autosomal dominant disorder caused by mutations in the TSC1 or TSC2 genes [152,153] that were associated with ASD in 17–63% of cases [154,155]. The authors observed that patients who develop ASD, compared with those who do not develop it, have an earlier age-at-seizure onset, more frequent seizures and a significantly greater amount of interictal epileptiform features in the left temporal lobe. This leads us to suppose that epileptiform SEAs may in some way contribute to determining the autistic phenotype, as proposed by El Achkar and Spence [156]. Moreover, some authors [17,145] maintain that epileptiform SEAs are significantly associated with abnormal development during the first year of life, and Romero-González et al., 2022 [150] believe that in preschool children, these abnormalities suggest worse development in ASD clinical features. Other authors [137,145] also maintain that epileptiform SEAs correlate with ASD phenotype severity, in particular, with stereotypies and aggressiveness. Nicotera et al., 2019 [145], state that these abnormalities are also associated with language impairment and intellectual disability, while Hrdlicka et al., 2003 [17] maintain that there is no significant correlation with the latter. Romero-González et al., 2022 [150] report that ASD patients with epileptiform SEAs present more affectation, particularly in the areas of prosocial behaviors and social relationships, and tend to exhibit lower adaptive functioning, higher scores on global executive functioning, ASD severity and total scores of coexisting psychiatric problems, even if they do not reach statistical significance. Regarding autistic regression, several authors [17,109,135,140] believe that there is no significant association with epileptiform SEAs but rather with epilepsy [17].

Given what has been reported, several hypotheses attempt to correlate epileptiform SEAs with the pathophysiology of ASD. Some authors maintain that epileptiform discharges, especially if early, with or without seizures, could have a negative impact on brain development, with consequent alteration of cognitive functions and behavior [157] and also social skills, relational abilities and inhibition control [150]. Hirosawa et al. believe, however, that epileptiform SEAs could have an ambivalent role in the pathophysiology of ASD. In their first study [158], they observed that a high number of epileptiform SEAs is associated with lower intelligence in non-ASD subjects and higher intelligence in ASD subjects. In their second study [159], they found better social skills in an ASD patient population with a high number of epileptiform SEAs: this association is supported by Hartley-McAndrew and Weinstock, 2010 [143] and contested by Milovanovic et al., 2019 [60]. In their third study [160], Hirosawa et al., 2021 formulated the hypothesis of the ambivalent nature of epileptiform SEAs: they claim that epileptiform SEAs could have the ability to “normalize” the neuroatypical development of ASD patients, lowering ASD severity; however, when the effect extends beyond brain tolerance, epileptiform SEAs could actually worsen autistic phenotype. Nonetheless, it is always necessary to keep in mind that the results they obtained are limited by the fact that all healthy controls selected for the study never presented SEAs.

In conclusion, given the potential pathophysiological role that EEG abnormalities, especially in the temporal and frontal lobes, could play in ASD, further study of cerebral electrophysiology in ASD patients is needed. In fact, EEG abnormalities, in addition to constituting a potential tool for early diagnosis—given their interesting relationship with a child’s development during the first year of life [17,145]—could also provide useful prognostic information [160]. Nonetheless, it is important to remember that ASD is a multifactorial disorder and its origin is not fully known, to the extent that idiopathic autism still represents 80–90% of all diagnoses [8,161,162,163,164]. The hypothesis of the etiopathogenetic link between epileptic seizures and autism can be advanced, at the moment, only for some patients, taking as a model syndromic forms of ASD in which epilepsy and autism often co-occur, such as, for example, Rett Syndrome [165], Angelman Syndrome [166] and Fragile X Syndrome [167].

### 4.4. Roles of EEG Recording Techniques and Sleep in Studying ASD

Continuing with the subject of heterogeneity, in addition to the extreme variability in the EEG equipment used, sometimes not even specified, another crucial element is constituted by the use of activation tests, which, in the articles we selected, are represented by hyperventilation (HV) [168], intermittent photic stimulation (IPS) [169] and sleep deprivation [170,171].

These activation procedures therefore allow us to increase the probability of finding EEG abnormalities and, consequently, permit us to increase, albeit in a limited number of patients, especially young ones, the overall number of different types of identifiable EEG abnormalities [172]. Although HV and IPS are activation procedures recommended as standard in routine and sleep EEG [173], among the articles we selected, only three of them mention their application [56,59,60], while only two studies mention sleep deprivation [55,59]: in one of these [59] sleep deprivation was not applied to all patients in the cohort, but the actual number was not made explicit. In the remaining six articles, therefore, the rate of EEG abnormalities is probably underestimated, as a portion was not detected with the use of activation procedures: this inevitably affects the comparability of the results obtained.

Similar considerations can be made for the duration of EEG recordings: the longer the recording, the greater the probability of finding EEG abnormalities. In routine EEG, it is true that the majority of abnormalities can be found during the first 20 min of recording, but it has been observed that it is possible to increase the yield by 11% by extending the duration to 40 min. Sometimes it is not possible to routinely carry out very long recordings due to costs, but from a research point of view, this aspect has a non-negligible weight, as the results obtained from recordings of different durations are not comparable in a standardized manner to each other. In the articles we selected, a very notable heterogeneity in duration can be observed, both between different cohorts and within the same cohort: one article reports a duration of 20 min [60], one of 30 min [59], two of ≥30 min [56,57], one of 40–60 min [55], one of 60 min [61], one of 40–70 min [54] and one of 30 min for awake-EEG and 60 min for sleep-EEG; in two cases, the duration is not made explicit [58,63]. Furthermore, in studies in which the duration of recording is provided as a range, the actual number of patients who underwent recordings of different durations is not specified.

Finally, another important aspect is represented by the resting state condition (wakefulness and/or sleep) in which the EEG recordings were carried out: once again, a significant heterogeneity can be observed, both between different cohorts and within the same cohort, as previously stated. In this case, however, the recordings were not carried out in the same resting state, invalidating the standardization of the comparison, and also for some patients, a sleep recording was not obtained, which is extremely relevant because it decreases the probability of identifying EEG abnormalities [137,141,145,174]. Furthermore, providing an overnight EEG recording would allow us to analyze complete sleep cycles, including REM sleep, and could provide additional stronger information on the characterization of EEG abnormalities and their possible correlation with the ASD phenotype. Unfortunately, an overnight study on a child with ASD tends to be quite challenging, limited by the poor cooperation of patients. Nevertheless, as summarized by Petruzzelli et al., 2021 [175], in the last two decades, some scientific studies tried to examine objective macro- and microstructural sleep parameters by performing polysomnography or sleep EEG overnight. The study by Petruzzelli et al., 2021 provided a quantitative analysis of sleep microstructure patterns and showed alteration in sleep spindles, cycling alternating patterns, band powers and the Mu rhythm in ASD patients. However, the significance of these findings should be approached with caution due to the limited number of studies in this field and the clinical diversity of the study cohorts. On the other hand, regarding macrostructural sleep parameters, significant findings in ASD patients showed that ASD children take longer to fall asleep, get less sleep and experience more awakenings after falling asleep compared with typically developing children, which corresponds to a higher prevalence of nighttime insomnia symptoms [175].

The relationship between ASD and sleep has always aroused much interest, primarily due to the greater risk of sleep disorders in these patients, compared with the neurotypical population, first and foremost, insomnia [176]. In ASD children, these disorders have a prevalence variably reported in the literature between 60% and 90% [177,178,179,180,181,182]: this variability could be due, at least in part, to the confounding action carried out by some psychiatric comorbidities that we often find in ASD patients, such as Anxiety Disorders, Mood Disorders and ADHD, which can interfere with sleep and alter the presentation of sleep disorders [183,184,185]. It has been proposed that the ASD-sleep disorder association recognizes a possible common neurophysiopathological basis [186,187]: in patients suffering from chronic insomnia, some authors [188] found a reduction in GABAergic transmission, which, as already discussed, is the basis of one of the most accredited pathophysiological theories regarding ASD. More recently, it has been observed that physiologically, the excitation/inhibition (E/I) balance changes dynamically in a sleep-dependent manner over the course of 24 h [189]: in particular, GABAergic transmission is reduced during NREM sleep, leading to an increase in the E/I ratio in this phase [190]. Therefore, it is reasonable to assume that the alterations in physiological sleep, which we often find in ASD patients, can in turn alter the physiological regulation of the E/I balance, intervening in some way in the pathophysiology of ASD. In this regard, in a recent study [186], the authors proposed three possible hypotheses that try to explain the potential relationship between ASD and sleep disorders:

- According to hypothesis 1, there would be a causal relationship between ASD and an E/I imbalance, while sleep disorders would not be associated with either ASD or an E/I imbalance;
- According to hypothesis 2, there is no known causal relationship between ASD and an E/I imbalance, which could be more adequately investigated once the confounding factor, constituted by sleep disorders, has been removed;
- According to hypothesis 3, there would be a bidirectional pathophysiological relationship between ASD and sleep disorders, which in turn is causally associated with an E/I imbalance.

Consequently, further studies are needed to resolve the conflict between them, but considering the third hypothesis as being ideally correct, further analysis of the E/I balance during sleep could help us to better understand the pathophysiology underlying ASD [186].

The EEG tracings of ASD patients recorded during sleep are, indeed, full of abnormalities, which, in some cases, show a certain association with the autism phenotype. Six articles [54,55,56,58,60,61] among those we selected expose the abnormalities found during sleep, which include both epileptiform and non-epileptiform ones. Among the former, we find spikes, polyspikes, polyspike waves, slow waves, slow spike waves, sharp waves and spike–wave complexes; among the latter, we find background rhythmic theta/delta slowing, generalized intermittent slow waves, excessive beta activity, asymmetry, dysrhythmia/slowing down of the ground rhythm, paradoxical delta activity, irregular background activity, asynchrony and abnormal fast activity. Only Milovanovic et al., 2019 [60] and Kammoun et al., 2022 [55] also report the presence of disorganization of the sleep architecture, although this aspect is widely reported in numerous studies in the literature [191,192,193,194,195]. Kammoun et al., 2022 [55], in particular, report the presence of asynchrony of sleep spindles and poorly organized EEG and also suggest that sleep disorganization shows a certain association with language regression and behavioral problems. This association with the phenotype has been the subject of several studies in the literature that have resulted in contradictory conclusions: according to some authors, there is no association [109,196], while according to others, a relationship can be inferred [180,191]. Studies conducted on an animal model [197,198] have shown that the mutations responsible for the c-terminal truncation of Shank3 (Shank3∆C) induce the appearance of sleep alterations. Shank3ΔC mice sleep less than controls, take longer to fall asleep and exhibit EEG abnormalities, represented by reduced EEG slow-wave delta (0.5–4 Hz) activity in NREM sleep [197] and reduced delta activity accompanied by an increase in theta activity in REM sleep [198]. The reduction in the power in the delta range in NREM sleep worsens with age, similar to how sleep disorders occur in ASD patients [198]. Interestingly, mutations responsible for the c-terminal truncation of Shank3 were also found in human ASD patients, in which they were associated with some phenotypic characteristics, including the absence of reciprocal social interaction, the absence of interest toward other children, repetitive behaviors, restricted interests, language regression and deficits in imitation, pretend play and symbolic play [199]. This appears to be extremely important data, also considering that sleep disorders seem to be associated with increased restricted and repetitive behaviors [200].

Despite recent progress, there is still no certainty about the relationship between ASD and sleep disorders. For this reason, it is important to carry out studies on sleep EEG recordings in ASD patients and continue to investigate the signs and symptoms of sleep disorders because they are often identified before the diagnosis of ASD and could, according to some, constitute *core* symptoms of ASD [201].

### 4.5. Age as a Key to Interpret EEG Tracings in ASD Patients

Finally, another non-negligible aspect concerns the age of patients who form the cohorts of the studies covered by this review.

The articles selected cover overall the entire age range between 1.4 years and 12 years, with a prevailing interest in the middle childhood (6–11 y) and young teen (12–14 y) groups [202]. On the contrary, only two studies focus only on patients aged 5 years or less [54,57]. This reflects a fairly common trend in the literature, which is not in favor of the research work of an EEG biomarker for early diagnosis. Today, ASD is widely considered a Connectopathy [92], controversially characterized by hypo- or hyper-connectivity [203], depending on different studies [204]. This controversy was subsequently resolved by demonstrating the co-occurrence of the two phenomena in different areas of the brain [205] and by hypothesizing the coexistence between long-range hypoconnectivity and local hyperconnectivity [206]. It has been observed that these abnormalities of functional connectivity correlate with growth, as hyperconnectivity tends to prevail in childhood, while hypoconnectivity makes its appearance in adolescents/adults, alone [207], or in combination with hyperconnectivity [208]. Connectivity is studied with the use of either resting state functional MRI or diffusion tensor imaging [209], but it is plausible that these age-related differences in brain connectivity could be responsible for different electrophysiological brain behaviors in preschool children and adolescents. In addition, among ASD subjects, epilepsy has a higher peak incidence in adolescence [24]; therefore, it is inevitable that the pool of epileptiform EEG abnormalities will tend to be larger among older individuals. For these reasons, in order to be able to identify a specific EEG biomarker for the early diagnosis of ASD, it is appropriate to conduct studies that focus their attention more selectively on the preschool population.

Lastly, as far as gender is concerned, the ten studies we selected are all characterized by a profound disparity: in particular, the M:F ratio ranges between a minimum of 1.3:1 [63] and a maximum of 5.6:1 [54], with an average of 2.95:1. These data are in line with the M:F ratio in the prevalence of ASD.

## 5. Conclusions

The heterogeneity highlighted in the articles we selected undoubtedly poses some limitations, which are fully explored in this review, but, at the same time, allows us to draw some conclusions that could guide future studies:

- It is important to investigate an EEG during sleep, with a preference for, in particular, prolonged recordings, as they allow for the identification of potentially significant EEG abnormalities with greater probability [17,137];
- Since EEG abnormalities show an interesting association with the ASD phenotype, in particular, with the degree of severity [55,58] and also with developmental delay [54], IQ [59,61] and behavioral disorders [137], studying abnormal brain electrical activity could provide valuable help to understand better the pathophysiology underlying ASD.

Ultimately, EEG may have a future value from a prognostic, therapeutic and diagnostic point of view, especially with regard to the development of an early suspicion of ASD. It is desirable, and also necessary, to carry out further studies in this area, especially selectively on preschoolers, in order to overcome the non-specificity of the results obtained so far and aim toward the identification of a possible EEG biomarker of ASD.

## Figures and Tables

**Table 1:** General data of the selected articles, demographics of the cohorts, EEG findings and functional profile of the patients.

AUTHORS (Publication Year)	ASD DIAGNOSTIC CRITERIA	AGE RANGE/MEAN AGE (Years) with Standard Deviation (SD) Where Specified	EEG RECORDING FEATURES	TYPE (Epileptiform/ Non-Epileptiform)	NUMBER OF PATIENTS WITH EPILEPSY	FUNCTIONAL PROFILE AMONG PATIENTS WITH ABNORMAL EEG
DATABASE	PATIENT COHORT (M/F)			LOCATION		
Kanemura H. et al. (2013) [63]	DSM-IV *n* = 21 (12/9)	9–12/10.25 (at the last observation)	- Awake/sleep: not specified. - Technical details: digital EEG. - Duration: not specified. - Activation procedures: not specified.	Epileptiform abnormalities (*n* = 11)	6	Not specified
Pubmed/Google Scholar	*n* = 21 (12/9)			Focal and multifocal		
Barbosa de Matos M. et al. (2015) [57]	DSM-5	1.42–4.83/ASD diagnosis 2.99 ± 0.98 and epilepsy diagnosis 2.80 ± 0.36	- Awake/sleep: Not specified. - Technical details: 10–20 system with digital equipment. - Duration: ≥30 min. - Activation procedures: not specified.	Epileptiform abnormalities (*n* = 9) Disorganized background activity (*n* = 28)	11	Not specified
Google Scholar	*n* = 63 (40/23)			Focal, multifocal and generalized		
Yousef A. M. et al. (2017) [58]	DSM IV Childhood Autism Rating Scale (CARS) > 30	2–12/4.97 ± 2.9 SD	- Awake/sleep: only sleep (induced).	Epileptiform abnormalities (*n* = 20)	0	Direct correlation between ASD severity (CARS) and EEG abnormalities (predominantly generalized discharges)
	*n* = 40 (28/12)					
Google Scholar				Focal and generalized		
Anukirthiga B. et al. (2019) [59]	DSM-5	6–12/7.7	- Awake/sleep: awake–sleep not in all children (Triclofos sedation for non-cooperative patients). - Technical details: child and referential montages with 10–20 system with digital EEG machine. - Duration: 30 min. - Activation procedures: hyperventilation and photic stimulation, when possible, and sleep deprivation.	Epileptiform abnormalities (*n* = 41)	Among ASD patients: 41 (24 with IEDs)	Concomitant epilepsy and IEDs
Pubmed/Scopu/Google Scholar	*n* = 130 of which 90 with ASD (69/21)			Focal, multifocal and generalized		
Milovanovic M. et al. (2019) [60]	ICD-10 Autism Diagnostic Interview-Revised (ADI-R)	2–18/6.58 ± 3.72 SD	- Awake/sleep: awake (98)–sleep (46) (eventually induced with melatonin). - Technical details: electro-capswere with 10–20 system with digital EEG. - Duration: 20 min. - Activation procedures: 5 min of hyperventilation and photo stimulation.	Epileptiform abnormalities (*n* = 31) Abnormal background activity, focal slow activity and abnormal sleep architecture (*n* = 19)	17 (all of them with epileptiform abnormalities)	No evidence of direct correlation between adaptive behavior and epilepsy/EEG abnormalities
Pubmed/Scopus/Google Scholar	*n* = 112 (90/22)			Focal and generalized		
Akhter S. (2021) [61]	DSM-IV-TR	2–12/5.25 ± 2.75	- Awake/sleep: sleep only. - Technical details: 10–20 system - Duration: 1 h. - Activation procedures: not specified.	Epileptiform abnormalities (*n* = 19) Background rhythmic theta/delta slowing, generalised intermittent slow waves, excessive beta activity and lateralized asymmetry (*n* = 8)	10	Variable correlation between EEG abnormalities and ID severity
Google Scholar	*n* = 52 (41/11)			Focal, multifocal and generalized		
Polat I. et al. (2022) [56]	DSM-5	2–17/boys 6.3 ± 3.7 girls 5.7 ± 3	- Awake/sleep: awake–sleep - Technical details: 10–20 system with digital EEG. - Duration: ≥30 min. - Activation procedures: hyperventilation and intermittent photic stimulation.	Epileptiform abnormalities (*n* = 37) Dysrhythmia/slowing down of the ground rhythm and paradoxical delta activity (*n* = 20)	25	Correlation between EEG abnormalities and functional profile not specified
Google Scholar	*n* = 166 (124/42)			Focal and generalized		
Sharma V. et al. (2022) [62]	DSM-5	3–14/5.6 ± 2.4	- Awake/sleep: awake only and awake–sleep. - Technical details: 10–20 system with digital tracing monitor and recorded with bipolar and referential montages. - Duration: 30 min if awake and 1 h if asleep. - Activation procedures: not specified.	Epileptiform abnormalities (*n* = not specified) Focal slowing and intermittent arrhythmic delta slowing (*n* = not specified)	23 (15 with EEG abnormalities)	Direct correlation between global developmental delay/ID severity and EEG abnormalities
Pubmed/Scopus/Google Scholar	*n* = 100 (80/20)			Focal, multifocal and generalized		
Kammoun I. et al. (2022) [55]	DSM-5	2–6/5	- Awake/sleep: sleep only. - Technical details: digital EEG system and data were referenced according to the longitudinal bipolar montage of the original eight electrode signals. - Duration: 40–60 min - Activation procedures: mild sleep deprivation (awaking 2–4 h prior to regular morning arousal).	Epileptiform abnormalities (*n* = not specified) Sleep disorganization (*n* = 5)	0	Direct correlation between EEG abnormalities and the severity of neurodevelopmental disorders
Google Scholar	*n* = 39 (23/16)			Focal and multifocal		
Santarone M. E. et al. (2023) [54]	DSM-5 ADOS-2 Italian Version ADI-R Italian Version	1.57–4.72/2.88	- Awake/sleep: awake–sleep. - Technical details: 10–20 system with digital EEG + ≥ two EMG electrodes. - Duration: 40–70 min. - Activation procedures: not specified.	Epileptiform abnormalities (*n* = not specified) Slow or irregular background activity, asymmetry, abnormal fast activity and asynchrony (*n* = not specified)	1	Direct correlation between nonepileptiform abnormalities during sleep and developmental delay
Pubmed/Scopus/Google Scholar	*n* = 292 (248/44)			Focal and generalized		

ASD: Autism Spectrum Disorder; EEG: electroencephalogram; DSM: Diagnostic and Statistical Manual of Mental Disorders; CARS: Childhood Autism Rating Scale; IQ: intelligence quotient; IEDs: interictal epileptiform discharges; ICD: International Classification of Diseases; ADI-R: Autism Diagnostic Interview—Revised; DSM-TR: Diagnostic and Statistical Manual of Mental Disorders—Text Revision; ID: intellectual disability.

## Data Availability

No new data were created or analyzed in this study. Data sharing is not applicable to this article.

## Abbreviations

ASD	Autism Spectrum Disorde r
EEG	Electroencephalogram
NDD	neurodevelopmental disorder
ID	intellectual disability
IQ	intelligence quotient
Glu	glutamate
GABA	gamma-aminobutyric acid
GABARD	delta-subunit of GABA-A receptors
TSC	Tuberous Sclerosis Complex
DSM	Diagnostic and Statistical Manual of Mental Disorders
ICD	International Classification of Diseases
qEEG	quantitative Eelectroencephalogram
DSM-TR	Diagnostic and Statistical Manual of Mental Disorders-Text Revision
ADOS-2	Autism Diagnostic Observation Schedule—Second Edition
ADI-R	Autism Diagnostic Interview—Revised
CARS	Childhood Autism Rating Scale
IEDs	interictal epileptic discharges
TLE	Temporal Lobe Epilepsy
GAD	glutamate decarboxylase
PV	Parvalbumin
PNS	Peripheral Nervous System
CNS	Central Nervous System
SEA	subclinical EEG abnormalities
FLE	Frontal Lobe Epilepsy
HV	hyperventilation
IPS	intermittent photic stimulation
ADHD	Attention-Deficit/Hyperactivity Disorder
NREM	non-rapid eye movement
REM	rapid eye movement

## References

[1] American Psychiatric Association (2022). Diagnostic and Statistical Manual of Mental Disorders: DSM-5-TR.

[2] Zeidan J., Fombonne E., Scorah J., Ibrahim A., Durkin M.S., Saxena S., Yusuf A., Shih A., Elsabbagh M. (2022). Global Prevalence of Autism: A Systematic Review Update. Autism Res..

[3] Maenner M.J., Warren Z., Williams A.R., Amoakohene E., Bakian A.V., Bilder D.A., Durkin M.S., Fitzgerald R.T., Furnier S.M., Hughes M.M. (2023). Prevalence and Characteristics of Autism Spectrum Disorder among Children Aged 8 Years—Autism and Developmental Disabilities Monitoring Network, 11 Sites, United States, 2020. MMWR Surveill. Summ..

[4] Dewey D. (2018). What Is Comorbidity and Why Does It Matter in Neurodevelopmental Disorders?. Curr. Dev. Disord. Rep..

[5] Al-Beltagi M. (2021). Autism Medical Comorbidities. World J. Clin. Pediatr..

[6] Lord C., Elsabbagh M., Baird G., Veenstra-Vanderweele J. (2018). Autism Spectrum Disorder. Lancet.

[7] Emberti Gialloreti L., Mazzone L., Benvenuto A., Fasano A., Alcon A.G., Kraneveld A., Moavero R., Raz R., Riccio M.P., Siracusano M. (2019). Risk and Protective Environmental Factors Associated with Autism Spectrum Disorder: Evidence-Based Principles and Recommendations. J. Clin. Med..

[8] National Institute of Health (2019). About Autism.

[9] Won H., Mah W., Kim E. (2013). Autism Spectrum Disorder Causes, Mechanisms, and Treatments: Focus on Neuronal Synapses. Front. Mol. Neurosci..

[10] Khoja S., Haile M.T., Chen L.Y. (2023). Advances in Neurexin Studies and the Emerging Role of Neurexin-2 in Autism Spectrum Disorder. Front. Mol. Neurosci..

[11] Azhari A., Truzzi A., Neoh M.J.-Y., Balagtas J.P.M., Tan H.H., Goh P.P., Ang X.A., Setoh P., Rigo P., Bornstein M.H. (2020). A Decade of Infant Neuroimaging Research: What Have We Learned and Where Are We Going?. Infant Behav. Dev..

[12] Britton J.W., Frey L.C., Hopp J.L., Korb P., Koubeissi M.Z., Lievens W.E., Pestana-Knight E.M., St Louis E.K. (2016). Electroencephalography (EEG): An Introductory Text and Atlas of Normal and Abnormal Findings in Adults, Children, and Infants.

[13] Billeci L., Sicca F., Maharatna K., Apicella F., Narzisi A., Campatelli G., Calderoni S., Pioggia G., Muratori F. (2013). On the Application of Quantitative EEG for Characterizing Autistic Brain: A Systematic Review. Front. Hum. Neurosci..

[14] Boutros N.N., Lajiness-O’Neill R., Zillgitt A., Richard A.E., Bowyer S.M. (2015). EEG Changes Associated with Autistic Spectrum Disorders. Neuropsychiatr. Electrophysiol..

[15] Tuchman R., Cuccaro M., Alessandri M. (2010). Autism and Epilepsy: Historical Perspective. Brain Dev..

[16] Lukmanji S., Manji S.A., Kadhim S., Sauro K.M., Wirrell E.C., Kwon C.-S., Jetté N. (2019). The Co-Occurrence of Epilepsy and Autism: A Systematic Review. Epilepsy Behav..

[17] Hrdlicka M., Komarek V., Propper L., Kulisek R., Zumrova A., Faladova L., Havlovicova M., Sedlacek Z., Blatny M., Urbanek T. (2004). Not EEG Abnormalities but Epilepsy Is Associated with Autistic Regression and Mental Functioning in Childhood Autism. Eur. Child Adolesc. Psychiatry.

[18] Besag F.M.C., Vasey M.J. (2020). Seizures and Epilepsy in Autism Spectrum Disorder. Child Adolesc. Psychiatr. Clin..

[19] Ewen J.B., Marvin A.R., Law K., Lipkin P.H. (2019). Epilepsy and Autism Severity: A Study of 6975 Children. Autism Res..

[20] Lee B.H., Smith T., Paciorkowski A.R. (2015). Autism Spectrum Disorder and Epilepsy: Disorders with a Shared Biology. Epilepsy Behav..

[21] Jeste S.S., Tuchman R. (2015). Autism Spectrum Disorder and Epilepsy: Two Sides of the Same Coin?. J. Child Neurol..

[22] Zarakoviti E., Shafran R., Skuse D., McTague A., Batura N., Palmer T., Dalrymple E., Bennett S.D., Reilly C. (2023). Factor Associated with the Occurrence of Epilepsy in Autism: A Systematic Review. J. Autism Dev. Disord..

[23] Spence S.J., Schneider M.T. (2009). The Role of Epilepsy and Epileptiform EEGs in Autism Spectrum Disorders. Pediatr. Res..

[24] Deykin E.Y., MacMahon B. (1979). The Incidence of Seizures among Children with Autistic Symptoms. Am. J. Psychiatry.

[25] Viscidi E.W., Triche E.W., Pescosolido M.F., McLean R.L., Joseph R.M., Spence S.J., Morrow E.M. (2013). Clinical Characteristics of Children with Autism Spectrum Disorder and Co-Occurring Epilepsy. PLoS ONE.

[26] Amiet C., Gourfinkel-An I., Bouzamondo A., Tordjman S., Baulac M., Lechat P., Mottron L., Cohen D. (2008). Epilepsy in Autism Is Associated with Intellectual Disability and Gender: Evidence from a Meta-Analysis. Biol. Psychiatry.

[27] Calderoni S. (2023). Sex/Gender Differences in Children with Autism Spectrum Disorder: A Brief Overview on Epidemiology, Symptom Profile, and Neuroanatomy. J. Neurosci. Res..

[28] Polyak A., Rosenfeld J.A., Girirajan S. (2015). An Assessment of Sex Bias in Neurodevelopmental Disorders. Genome Med..

[29] Marín O. (2012). Interneuron Dysfunction in Psychiatric Disorders. Nat. Rev. Neurosci..

[30] Jiang X., Lachance M., Rossignol E. (2016). Involvement of Cortical Fast-Spiking Parvalbumin-Positive Basket Cells in Epilepsy. Prog. Brain Res..

[31] Bozzi Y., Provenzano G., Casarosa S. (2018). Neurobiological Bases of Autism-Epilepsy Comorbidity: A Focus on Excitation/Inhibition Imbalance. Eur. J. Neurosci..

[32] Leonzino M., Busnelli M., Antonucci F., Verderio C., Mazzanti M., Chini B. (2016). The Timing of the Excitatory-to-Inhibitory GABA Switch Is Regulated by the Oxytocin Receptor via KCC2. Cell Rep..

[33] Sarlo G.L., Holton K.F. (2021). Brain Concentrations of Glutamate and GABA in Human Epilepsy: A Review. Seizure.

[34] Akyuz E., Polat A.K., Eroglu E., Kullu I., Angelopoulou E., Paudel Y.N. (2021). Revisiting the Role of Neurotransmitters in Epilepsy: An Updated Review. Life Sci..

[35] Medina-Ceja L., García-Barba C. (2017). The Glutamate Receptor Antagonists CNQX and MPEP Decrease Fast Ripple Events in Rats Treated with Kainic Acid. Neurosci. Lett..

[36] Peret A., Christie L.A., Ouedraogo D.W., Gorlewicz A., Epsztein J., Mulle C., Crépel V. (2014). Contribution of Aberrant GluK2-Containing Kainate Receptors to Chronic Seizures in Temporal Lobe Epilepsy. Cell Rep..

[37] Rogawski M.A. (2013). AMPA Receptors as a Molecular Target in Epilepsy Therapy. Acta Neurol. Scand..

[38] Hussman J.P. (2001). Suppressed GABAergic Inhibition as a Common Factor in Suspected Etiologies of Autism. J. Autism Dev. Disord..

[39] Fatemi S.H., Reutiman T.J., Folsom T.D., Rooney R.J., Patel D.H., Thuras P.D. (2010). mRNA and Protein Levels for GABAAalpha4, Alpha5, Beta1 and GABABR1 Receptors Are Altered in Brains from Subjects with Autism. J. Autism Dev. Disord..

[40] Lawrence Y.A., Kemper T.L., Bauman M.L., Blatt G.J. (2010). Parvalbumin-, Calbindin-, and Calretinin-Immunoreactive Hippocampal Interneuron Density in Autism. Acta Neurol. Scand..

[41] Tyzio R., Nardou R., Ferrari D.C., Tsintsadze T., Shahrokhi A., Eftekhari S., Khalilov I., Tsintsadze V., Brouchoud C., Chazal G. (2014). Oxytocin-Mediated GABA Inhibition during Delivery Attenuates Autism Pathogenesis in Rodent Offspring. Science.

[42] Ben-Ari Y. (2015). Is Birth a Critical Period in the Pathogenesis of Autism Spectrum Disorders?. Nat. Rev. Neurosci..

[43] Ahring P.K., Liao V.W.Y., Gardella E., Johannesen K.M., Krey I., Selmer K.K., Stadheim B.F., Davis H., Peinhardt C., Koko M. (2022). Gain-of-Function Variants in GABRD Reveal a Novel Pathway for Neurodevelopmental Disorders and Epilepsy. Brain.

[44] Nebel R.A., Zhao D., Pedrosa E., Kirschen J., Lachman H.M., Zheng D., Abrahams B.S. (2016). Reduced CYFIP1 in Human Neural Progenitors Results in Dysregulation of Schizophrenia and Epilepsy Gene Networks. PLoS ONE.

[45] De Rubeis S., Pasciuto E., Li K.W., Fernández E., Di Marino D., Buzzi A., Ostroff L.E., Klann E., Zwartkruis F.J.T., Komiyama N.H. (2013). CYFIP1 Coordinates mRNA Translation and Cytoskeleton Remodeling to Ensure Proper Dendritic Spine Formation. Neuron.

[46] Parenti I., Lehalle D., Nava C., Torti E., Leitão E., Person R., Mizuguchi T., Matsumoto N., Kato M., Nakamura K. (2021). Missense and Truncating Variants in CHD5 in a Dominant Neurodevelopmental Disorder with Intellectual Disability, Behavioral Disturbances, and Epilepsy. Hum. Genet..

[47] Rodenas-Cuadrado P., Pietrafusa N., Francavilla T., La Neve A., Striano P., Vernes S.C. (2016). Characterisation of CASPR2 Deficiency Disorder--a Syndrome Involving Autism, Epilepsy and Language Impairment. BMC Med. Genet..

[48] De Ridder J., Verhelle B., Vervisch J., Lemmens K., Kotulska K., Moavero R., Curatolo P., Weschke B., Riney K., Feucht M. (2021). Early Epileptiform EEG Activity in Infants with Tuberous Sclerosis Complex Predicts Epilepsy and Neurodevelopmental Outcomes. Epilepsia.

[49] Domańska-Pakieła D., Kaczorowska M., Jurkiewicz E., Kotulska K., Dunin-Wąsowicz D., Jóźwiak S. (2014). EEG Abnormalities Preceding the Epilepsy Onset in Tuberous Sclerosis Complex Patients—A Prospective Study of 5 Patients. Eur. J. Paediatr. Neurol..

[50] Wu J.Y., Goyal M., Peters J.M., Krueger D., Sahin M., Northrup H., Au K.S., O’Kelley S., Williams M., Pearson D.A. (2019). Scalp EEG Spikes Predict Impending Epilepsy in TSC Infants: A Longitudinal Observational Study. Epilepsia.

[51] Gradisnik P., Zagradisnik B., Palfy M., Kokalj-Vokac N., Marcun-Varda N. (2015). Predictive Value of Paroxysmal EEG Abnormalities for Future Epilepsy in Focal Febrile Seizures. Brain Dev..

[52] Kanemura H., Mizorogi S., Aoyagi K., Sugita K., Aihara M. (2012). EEG Characteristics Predict Subsequent Epilepsy in Children with Febrile Seizure. Brain Dev..

[53] Wirrell E.C. (2010). Prognostic Significance of Interictal Epileptiform Discharges in Newly Diagnosed Seizure Disorders. J. Clin. Neurophysiol..

[54] Santarone M.E., Zambrano S., Zanotta N., Mani E., Minghetti S., Pozzi M., Villa L., Molteni M., Zucca C. (2023). EEG Features in Autism Spectrum Disorder: A Retrospective Analysis in a Cohort of Preschool Children. Brain. Sci..

[55] Kammoun I., BenTouhemi D., Hadjkacem I., Zouari H., Kamoun F., Khemekhem K., Ayadi H., Ellouze E., Hsairi I., Ghribi F. (2022). Autism Spectrum Disorder and Eeg Specificity: A Cross—Sectional Tunisian Study Specificite De L’eeg Dans Le Trouble Du Spectre Autistique: Une Etude Transversale Tunisienne. J. L’inf. Méd. Sfax.

[56] Polat İ., Has A.S., Yiş U., Ayanoğlu M., Okur D., Bayram E., Baykara H.B. (2022). Epilepsy and Electroencephalographic Abnormalities in Children with Autistic Spectrum Disorder. J. Dr Behcet Uz Child. Hosp..

[57] Barbosa de Matos M., Nau A.L., Fezer G.F., Zeigelboim B.S., Liberalesso P.B.N. (2015). Epilepsy and eeg abnormalities in children with autism spectrum disorder. J. Epilepsy Clin. Neurophysiol..

[58] Yousef A.M., Youssef U.M., El-Shabrawy A., Fattah N.R.A., Khedr H. (2017). EEG Abnormalities and Severity of Symptoms in Non-Epileptic Autistic Children. Egypt. J. Psychiatry.

[59] Anukirthiga B., Mishra D., Pandey S., Juneja M., Sharma N. (2019). Prevalence of Epilepsy and Inter-Ictal Epileptiform Discharges in Children with Autism and Attention-Deficit Hyperactivity Disorder. Indian J. Pediatr..

[60] Milovanovic M., Radivojevic V., Radosavljev-Kircanski J., Grujicic R., Toskovic O., Aleksić-Hil O., Pejovic-Milovancevic M. (2019). Epilepsy and Interictal Epileptiform Activity in Patients with Autism Spectrum Disorders. Epilepsy Behav..

[61] Akhter S., Shefa J., Mannan M. (2021). EEG Changes and Their Relationship with Intellectual Disability in Children with Autism Spectrum Disorders in a Tertiary Care Hospital. J. Int. Child Neurol. Assoc..

[62] Sharma V., Saini A.G., Malhi P., Singhi P. (2022). Epilepsy and EEG Abnormalities in Children with Autism Spectrum Disorders. Indian J. Pediatr..

[63] Kanemura H., Sano F., Tando T., Sugita K., Aihara M. (2013). Can EEG Characteristics Predict Development of Epilepsy in Autistic Children?. Eur. J. Paediatr. Neurol..

[64] American Psychiatric Association (1994). Diagnostic and Statistical Manual of Mental Disorders.

[65] American Psychiatric Association (2000). Diagnostic and Statistical Manual of Mental Disorders.

[66] World Health Organization (WHO) (1993). The ICD-10 Classification of Mental and Behavioural Disorders.

[67] American Psychiatric Association (2013). Diagnostic and Statistical Manual of Mental Disorders.

[68] Jarero-Basulto J.J., Gasca-Martínez Y., Rivera-Cervantes M.C., Ureña-Guerrero M.E., Feria-Velasco A.I., Beas-Zarate C. (2018). Interactions between Epilepsy and Plasticity. Pharmaceuticals.

[69] Larner A.J. (1995). Axonal Sprouting and Synaptogenesis in Temporal Lobe Epilepsy: Possible Pathogenetic and Therapeutic Roles of Neurite Growth Inhibitory Factors. Seizure.

[70] Schmeiser B., Zentner J., Prinz M., Brandt A., Freiman T.M. (2017). Extent of Mossy Fiber Sprouting in Patients with Mesiotemporal Lobe Epilepsy Correlates with Neuronal Cell Loss and Granule Cell Dispersion. Epilepsy Res..

[71] Stringer J.L., Agarwal K.S., Dure L.S. (1997). Is Cell Death Necessary for Hippocampal Mossy Fiber Sprouting?. Epilepsy Res..

[72] McNamara J.O., Huang Y.Z., Leonard A.S. (2006). Molecular Signaling Mechanisms Underlying Epileptogenesis. Sci. STKE.

[73] Fairless R., Williams S.K., Diem R. (2019). Calcium-Binding Proteins as Determinants of Central Nervous System Neuronal Vulnerability to Disease. Int. J. Mol. Sci..

[74] Vizi S., Bagosi A., Krisztin-Péva B., Gulya K., Mihály A. (2004). Repeated 4-Aminopyridine Seizures Reduce Parvalbumin Content in the Medial Mammillary Nucleus of the Rat Brain. Mol. Brain Res..

[75] Rubenstein J.L.R., Merzenich M.M. (2003). Model of Autism: Increased Ratio of Excitation/Inhibition in Key Neural Systems. Genes Brain Behav..

[76] Vignoli A., Fabio R.A., La Briola F., Giannatiempo S., Antonietti A., Maggiolini S., Canevini M.P. (2010). Correlations between Neurophysiological, Behavioral, and Cognitive Function in Rett Syndrome. Epilepsy Behav..

[77] Chao H.-T., Chen H., Samaco R.C., Xue M., Chahrour M., Yoo J., Neul J.L., Gong S., Lu H.-C., Heintz N. (2010). Dysfunction in GABA Signalling Mediates Autism-like Stereotypies and Rett Syndrome Phenotypes. Nature.

[78] Godoy L.D., Prizon T., Rossignoli M.T., Leite J.P., Liberato J.L. (2022). Parvalbumin Role in Epilepsy and Psychiatric Comorbidities: From Mechanism to Intervention. Front. Integr. Neurosci..

[79] Permyakov E.A., Uversky V.N. (2022). What Is Parvalbumin For?. Biomolecules.

[80] Rupert D.D., Shea S.D. (2022). Parvalbumin-Positive Interneurons Regulate Cortical Sensory Plasticity in Adulthood and Development Through Shared Mechanisms. Front. Neural Circuits.

[81] Kawaguchi Y., Kubota Y. (1997). GABAergic Cell Subtypes and Their Synaptic Connections in Rat Frontal Cortex. Cereb. Cortex.

[82] Lee E., Lee J., Kim E. (2017). Excitation/Inhibition Imbalance in Animal Models of Autism Spectrum Disorders. Biol. Psychiatry.

[83] Filice F., Vörckel K.J., Sungur A.Ö., Wöhr M., Schwaller B. (2016). Reduction in Parvalbumin Expression Not Loss of the Parvalbumin-Expressing GABA Interneuron Subpopulation in Genetic Parvalbumin and Shank Mouse Models of Autism. Mol. Brain.

[84] Hashemi E., Ariza J., Rogers H., Noctor S.C., Martínez-Cerdeño V. (2017). The Number of Parvalbumin-Expressing Interneurons Is Decreased in the Prefrontal Cortex in Autism. Cereb. Cortex.

[85] Cummings J.L., Miller B.L. (2007). Conceptual and Clinical Aspects of the Frontal Lobes. The Human Frontal Lobes: Functions and Disorders.

[86] Cristofori I., Cohen-Zimerman S., Grafman J. (2019). Executive Functions. Handb. Clin. Neurol..

[87] Patel A., Biso G.M.N.R., Fowler J.B. (2023). Neuroanatomy, Temporal Lobe. StatPearls.

[88] Velásquez C., Goméz E., Martino J. (2018). Mapping Visuospatial and Self-Motion Perception Functions in the Left Parietal Lobe. Neurosurg. Focus.

[89] Rehman A., Al Khalili Y. (2023). Neuroanatomy, Occipital Lobe. StatPearls.

[90] Schultz R.T. (2005). Developmental Deficits in Social Perception in Autism: The Role of the Amygdala and Fusiform Face Area. Int. J. Dev. Neurosci..

[91] Baron-Cohen S., Belmonte M.K. (2005). Autism: A Window onto the Development of the Social and the Analytic Brain. Annu. Rev. Neurosci..

[92] Belmonte M.K., Allen G., Beckel-Mitchener A., Boulanger L.M., Carper R.A., Webb S.J. (2004). Autism and Abnormal Development of Brain Connectivity. J. Neurosci..

[93] Lagae L., Pauwels J., Monté C.P., Verhelle B., Vervisch I. (2001). Frontal Absences in Children. Eur. J. Paediatr. Neurol..

[94] Cohen H., Le Normand M.T. (1998). Language Development in Children with Simple-Partial Left-Hemisphere Epilepsy. Brain Lang..

[95] Boone K.B., Miller B.L., Rosenberg L., Durazo A., McIntyre H., Weil M. (1988). Neuropsychological and Behavioral Abnormalities in an Adolescent with Frontal Lobe Seizures. Neurology.

[96] Lassonde M., Sauerwein H.C., Jambaqué I., Smith M.L., Helmstaedter C. (2000). Neuropsychology of Childhood Epilepsy: Pre- and Postsurgical Assessment. Epileptic Disord..

[97] Prévost J., Lortie A., Nguyen D., Lassonde M., Carmant L. (2006). Nonlesional Frontal Lobe Epilepsy (FLE) of Childhood: Clinical Presentation, Response to Treatment and Comorbidity. Epilepsia.

[98] Nolan M.A., Redoblado M.A., Lah S., Sabaz M., Lawson J.A., Cunningham A.M., Bleasel A.F., Bye A.M.E. (2004). Memory Function in Childhood Epilepsy Syndromes. J. Paediatr. Child Health.

[99] Sinclair D.B., Wheatley M., Snyder T. (2004). Frontal Lobe Epilepsy in Childhood. Pediatr. Neurol..

[100] Nolan M.A., Redoblado M.A., Lah S., Sabaz M., Lawson J.A., Cunningham A.M., Bleasel A.F., Bye A.M.E. (2003). Intelligence in Childhood Epilepsy Syndromes. Epilepsy Res..

[101] Lee I.-C., Chen Y.-J., Lee H.-S., Li S.-Y. (2014). Prognostic Factors for Outcome in Pediatric Probable Lesional Frontal Lobe Epilepsy with an Unknown Cause (Cryptogenic). J. Child Neurol..

[102] Riva D., Avanzini G., Franceschetti S., Nichelli F., Saletti V., Vago C., Pantaleoni C., D’Arrigo S., Andreucci E., Aggio F. (2005). Unilateral Frontal Lobe Epilepsy Affects Executive Functions in Children. Neurol. Sci..

[103] Tangviriyapaiboon D., Traisathit P., Siripornpanich V., Suyakong C., Apikomonkon H., Homkham N., Thumronglaohapun S., Srikummoon P. (2022). Detection of Electroencephalographic Abnormalities and Its Associated Factors among Children with Autism Spectrum Disorder in Thailand. Healthcare.

[104] Fujii E., Mori K., Miyazaki M., Hashimoto T., Harada M., Kagami S. (2010). Function of the Frontal Lobe in Autistic Individuals: A Proton Magnetic Resonance Spectroscopic Study. J. Med. Investig..

[105] Ito A., Abe N., Fujii T., Ueno A., Koseki Y., Hashimoto R., Mugikura S., Takahashi S., Mori E. (2011). The Role of the Dorsolateral Prefrontal Cortex in Deception When Remembering Neutral and Emotional Events. Neurosci. Res..

[106] Hill E.L. (2004). Executive Dysfunction in Autism. Trends Cogn. Sci..

[107] Kim S.-Y., Choi U.-S., Park S.-Y., Oh S.-H., Yoon H.-W., Koh Y.-J., Im W.-Y., Park J.-I., Song D.-H., Cheon K.-A. (2015). Abnormal Activation of the Social Brain Network in Children with Autism Spectrum Disorder: An FMRI Study. Psychiatry Investig..

[108] Baird G., Charman T., Baron-Cohen S., Cox A., Swettenham J., Wheelwright S., Drew A. (2000). A Screening Instrument for Autism at 18 Months of Age: A 6-Year Follow-up Study. J. Am. Acad. Child Adolesc. Psychiatry.

[109] Baird G., Simonoff E., Pickles A., Chandler S., Loucas T., Meldrum D., Charman T. (2006). Prevalence of Disorders of the Autism Spectrum in a Population Cohort of Children in South Thames: The Special Needs and Autism Project (SNAP). Lancet.

[110] Bertrand J., Mars A., Boyle C., Bove F., Yeargin-Allsopp M., Decoufle P. (2001). Prevalence of Autism in a United States Population: The Brick Township, New Jersey, Investigation. Pediatrics.

[111] Bölte S., Poustka F. (2002). The Relation between General Cognitive Level and Adaptive Behavior Domains in Individuals with Autism with and without Co-Morbid Mental Retardation. Child Psychiatry Hum. Dev..

[112] Bölte S., Dziobek I., Poustka F. (2009). Brief Report: The Level and Nature of Autistic Intelligence Revisited. J. Autism Dev. Disord..

[113] Carlsson L.H., Norrelgen F., Kjellmer L., Westerlund J., Gillberg C., Fernell E. (2013). Coexisting Disorders and Problems in Preschool Children with Autism Spectrum Disorders. Sci. World J..

[114] Developmental Disabilities Monitoring Network Surveillance Year 2010 Principal Investigators, Centers for Disease Control and Prevention (CDC) (2014). Prevalence of Autism Spectrum Disorder among Children Aged 8 Years—Autism and Developmental Disabilities Monitoring Network, 11 Sites, United States, 2010. MMWR Surveill. Summ..

[115] Chakrabarti S., Fombonne E. (2005). Pervasive Developmental Disorders in Preschool Children: Confirmation of High Prevalence. Am. J. Psychiatry.

[116] Charman T., Pickles A., Simonoff E., Chandler S., Loucas T., Baird G. (2011). IQ in Children with Autism Spectrum Disorders: Data from the Special Needs and Autism Project (SNAP). Psychol. Med..

[117] de Bildt A., Sytema S., Kraijer D., Minderaa R. (2005). Prevalence of Pervasive Developmental Disorders in Children and Adolescents with Mental Retardation. J. Child Psychol. Psychiatry.

[118] Matson J.L., Shoemaker M. (2009). Intellectual Disability and Its Relationship to Autism Spectrum Disorders. Res. Dev. Disabil..

[119] Miller J.S., Bilder D., Farley M., Coon H., Pinborough-Zimmerman J., Jenson W., Rice C.E., Fombonne E., Pingree C.B., Ritvo E. (2013). Autism Spectrum Disorder Reclassified: A Second Look at the 1980s Utah/UCLA Autism Epidemiologic Study. J. Autism Dev. Disord..

[120] Jones C.R.G., Happé F., Pickles A., Marsden A.J.S., Tregay J., Baird G., Simonoff E., Charman T. (2011). “Everyday Memory” Impairments in Autism Spectrum Disorders. J. Autism Dev. Disord..

[121] Hajri M., Abbes Z., Yahia H.B., Jelili S., Halayem S., Mrabet A., Bouden A. (2022). Cognitive Deficits in Children with Autism Spectrum Disorders: Toward an Integrative Approach Combining Social and Non-Social Cognition. Front. Psychiatry.

[122] Woolfenden S., Sarkozy V., Ridley G., Coory M., Williams K. (2012). A Systematic Review of Two Outcomes in Autism Spectrum Disorder—Epilepsy and Mortality. Dev. Med. Child Neurol..

[123] Matsuo M., Maeda T., Ishii K., Tajima D., Koga M., Hamasaki Y. (2011). Characterization of Childhood-Onset Complex Partial Seizures Associated with Autism Spectrum Disorder. Epilepsy Behav..

[124] Bailey A., Le Couteur A., Gottesman I., Bolton P., Simonoff E., Yuzda E., Rutter M. (1995). Autism as a Strongly Genetic Disorder: Evidence from a British Twin Study. Psychol. Med..

[125] Mouridsen S.E., Rich B., Isager T. (1999). Epilepsy in Disintegrative Psychosis and Infantile Autism: A Long-Term Validation Study. Dev. Med. Child Neurol..

[126] Rossi P.G., Parmeggiani A., Bach V., Santucci M., Visconti P. (1995). EEG Features and Epilepsy in Patients with Autism. Brain Dev..

[127] Gillberg C. (1984). Autistic Children Growing up: Problems during Puberty and Adolescence. Dev. Med. Child Neurol..

[128] Volkmar F.R., Nelson D.S. (1990). Seizure Disorders in Autism. J. Am. Acad. Child Adolesc. Psychiatry.

[129] Gedye A. (1991). Frontal Lobe Seizures in Autism. Med. Hypotheses.

[130] Maurer R.G., Damasio A.R. (1982). Childhood Autism from the Point of View of Behavioral Neurology. J. Autism Dev. Disord..

[131] Ross E.D. (1981). The Aprosodias. Functional-Anatomic Organization of the Affective Components of Language in the Right Hemisphere. Arch. Neurol..

[132] Lee J., Lee K. (2021). Parvalbumin-Expressing GABAergic Interneurons and Perineuronal Nets in the Prelimbic and Orbitofrontal Cortices in Association with Basal Anxiety-like Behaviors in Adult Mice. Behav. Brain Res..

[133] Tuchman R.F., Rapin I. (1997). Regression in Pervasive Developmental Disorders: Seizures and Epileptiform Electroencephalogram Correlates. Pediatrics.

[134] Yasuhara A. (2010). Correlation between EEG Abnormalities and Symptoms of Autism Spectrum Disorder (ASD). Brain Dev..

[135] Parmeggiani A., Barcia G., Posar A., Raimondi E., Santucci M., Scaduto M.C. (2010). Epilepsy and EEG Paroxysmal Abnormalities in Autism Spectrum Disorders. Brain Dev..

[136] Akshoomoff N., Farid N., Courchesne E., Haas R. (2007). Abnormalities on the Neurological Examination and EEG in Young Children with Pervasive Developmental Disorders. J. Autism Dev. Disord..

[137] Mulligan C.K., Trauner D.A. (2014). Incidence and Behavioral Correlates of Epileptiform Abnormalities in Autism Spectrum Disorders. J. Autism Dev. Disord..

[138] Swatzyna R.J., Tarnow J.D., Turner R.P., Roark A.J., MacInerney E.K., Kozlowski G.P. (2017). Integration of EEG Into Psychiatric Practice: A Step toward Precision Medicine for Autism Spectrum Disorder. J. Clin. Neurophysiol..

[139] Valvo G., Baldini S., Brachini F., Apicella F., Cosenza A., Ferrari A.R., Guerrini R., Muratori F., Romano M.F., Santorelli F.M. (2013). Somatic Overgrowth Predisposes to Seizures in Autism Spectrum Disorders. PLoS ONE.

[140] Canitano R., Luchetti A., Zappella M. (2005). Epilepsy, Electroencephalographic Abnormalities, and Regression in Children with Autism. J. Child Neurol..

[141] Chez M.G., Chang M., Krasne V., Coughlan C., Kominsky M., Schwartz A. (2006). Frequency of Epileptiform EEG Abnormalities in a Sequential Screening of Autistic Patients with No Known Clinical Epilepsy from 1996 to 2005. Epilepsy Behav..

[142] Hara H. (2007). Autism and Epilepsy: A Retrospective Follow-up Study. Brain Dev..

[143] Hartley S.L., Barker E.T., Seltzer M.M., Floyd F., Greenberg J., Orsmond G., Bolt D. (2010). The Relative Risk and Timing of Divorce in Families of Children with an Autism Spectrum Disorder. J. Fam. Psychol..

[144] Capal J.K., Carosella C., Corbin E., Horn P.S., Caine R., Manning-Courtney P. (2018). EEG Endophenotypes in Autism Spectrum Disorder. Epilepsy Behav..

[145] Nicotera A.G., Hagerman R.J., Catania M.V., Buono S., Di Nuovo S., Liprino E.M., Stracuzzi E., Giusto S., Di Vita G., Musumeci S.A. (2019). EEG Abnormalities as a Neurophysiological Biomarker of Severity in Autism Spectrum Disorder: A Pilot Cohort Study. J. Autism Dev. Disord..

[146] Eeg-Olofsson O., Petersén I., Selldén U. (1971). The Development of the Electroencephalogram in Normal Children from the Age of 1 through 15 Years. Paroxysmal Activity. Neuropadiatrie.

[147] Cavazzuti G.B., Cappella L., Nalin A. (1980). Longitudinal Study of Epileptiform EEG Patterns in Normal Children. Epilepsia.

[148] Capdevila O.S., Dayyat E., Kheirandish-Gozal L., Gozal D. (2008). Prevalence of Epileptiform Activity in Healthy Children during Sleep. Sleep Med..

[149] Hashimoto T., Sasaki M., Sugai K., Hanaoka S., Fukumizu M., Kato T. (2001). Paroxysmal Discharges on EEG in Young Autistic Patients Are Frequent in Frontal Regions. J. Med. Investig..

[150] Romero-González M., Navas-Sánchez P., Marín-Gámez E., Barbancho-Fernández M.A., Fernández-Sánchez V.E., Lara-Muñoz J.P., Guzmán-Parra J. (2022). EEG Abnormalities and Clinical Phenotypes in Pre-School Children with Autism Spectrum Disorder. Epilepsy Behav..

[151] Numis A.L., Major P., Montenegro M.A., Muzykewicz D.A., Pulsifer M.B., Thiele E.A. (2011). Identification of Risk Factors for Autism Spectrum Disorders in Tuberous Sclerosis Complex. Neurology.

[152] (1993). European Chromosome 16 Tuberous Sclerosis Consortium Identification and Characterization of the Tuberous Sclerosis Gene on Chromosome 16. Cell.

[153] van Slegtenhorst M., de Hoogt R., Hermans C., Nellist M., Janssen B., Verhoef S., Lindhout D., van den Ouweland A., Halley D., Young J. (1997). Identification of the Tuberous Sclerosis Gene TSC1 on Chromosome 9q34. Science.

[154] de Vries P.J., Hunt A., Bolton P.F. (2007). The Psychopathologies of Children and Adolescents with Tuberous Sclerosis Complex (TSC): A Postal Survey of UK Families. Eur. Child Adolesc. Psychiatry.

[155] Crucitti J., Hyde C., Enticott P.G., Stokes M.A. (2022). A Systematic Review of Frontal Lobe Volume in Autism Spectrum Disorder Revealing Distinct Trajectories. J. Integr. Neurosci..

[156] El Achkar C.M., Spence S.J. (2015). Clinical Characteristics of Children and Young Adults with Co-Occurring Autism Spectrum Disorder and Epilepsy. Epilepsy Behav..

[157] Lado F.A., Rubboli G., Capovilla G., Avanzini G., Moshé S.L. (2013). Pathophysiology of Epileptic Encephalopathies. Epilepsia.

[158] Hirosawa T., Kikuchi M., Fukai M., Hino S., Kitamura T., An K.-M., Sowman P., Takahashi T., Yoshimura Y., Miyagishi Y. (2018). Association between Magnetoencephalographic Interictal Epileptiform Discharge and Cognitive Function in Young Children with Typical Development and with Autism Spectrum Disorders. Front. Psychiatry.

[159] Hirosawa T., Sowman P.F., Fukai M., Kameya M., Soma D., Hino S., Kitamura T., An K.-M., Yoshimura Y., Hasegawa C. (2020). Relationship between Epileptiform Discharges and Social Reciprocity or Cognitive Function in Children with and without Autism Spectrum Disorders: An MEG Study. Psychiatry Clin. Neurosci..

[160] Hirosawa T., An K.-M., Soma D., Shiota Y., Sano M., Kameya M., Hino S., Naito N., Tanaka S., Yaoi K. (2021). Epileptiform Discharges Relate to Altered Functional Brain Networks in Autism Spectrum Disorders. Brain Commun..

[161] Faras H., Al Ateeqi N., Tidmarsh L. (2010). Autism Spectrum Disorders. Ann. Saudi Med..

[162] Ceman S., Saugstad J. (2011). MicroRNAs: Meta-Controllers of Gene Expression in Synaptic Activity Emerge as Genetic and Diagnostic Markers of Human Disease. Pharmacol. Ther..

[163] Herman G.E., Henninger N., Ratliff-Schaub K., Pastore M., Fitzgerald S., McBride K.L. (2007). Genetic Testing in Autism: How Much Is Enough?. Genet. Med..

[164] Geschwind D.H. (2011). Genetics of Autism Spectrum Disorders. Trends Cogn. Sci..

[165] D’Mello S.R. (2023). Rett and Rett-Related Disorders: Common Mechanisms for Shared Symptoms?. Exp. Biol. Med..

[166] Perrino P.A., Chamberlain S.J., Eigsti I.-M., Fitch R.H. (2021). Communication-Related Assessments in an Angelman Syndrome Mouse Model. Brain Behav..

[167] Stone W.L., Basit H., Shah M., Los E. (2023). Fragile X Syndrome. StatPearls.

[168] Mecarelli O. (2009). Manuale Teorico-Pratico di Elettroencefalografia (pp. 194–214). Medici Oggi.

[169] Bickford R.G., Sem-Jacobsen C.W., White P.T., Daly D. (1952). Some Observations on the Mechanism of Photic and Photometrazol Activation. Electroencephalogr. Clin. Neurophysiol..

[170] Pratt K.L., Mattson R.H., Weikers N.J., Williams R. (1968). EEG Activation of Epileptics Following Sleep Deprivation: A Prospective Study of 114 Cases. Electroencephalogr. Clin. Neurophysiol..

[171] Badawy R.A.B., Curatolo J.M., Newton M., Berkovic S.F., Macdonell R.A.L. (2006). Sleep Deprivation Increases Cortical Excitability in Epilepsy: Syndrome-Specific Effects. Neurology.

[172] Baldin E., Hauser W.A., Buchhalter J.R., Hesdorffer D.C., Ottman R. (2017). Utility of EEG Activation Procedures in Epilepsy: A Population-Based Study. J. Clin. Neurophysiol..

[173] Peltola M.E., Leitinger M., Halford J.J., Vinayan K.P., Kobayashi K., Pressler R.M., Mindruta I., Mayor L.C., Lauronen L., Beniczky S. (2023). Routine and Sleep EEG: Minimum Recording Standards of the International Federation of Clinical Neurophysiology and the International League Against Epilepsy. Epilepsia.

[174] Miskin C., Carvalho K.S., Valencia I., Legido A., Khurana D.S. (2015). EEG Duration: The Long and the Short of It. J. Child Neurol..

[175] Petruzzelli M.G., Matera E., Giambersio D., Marzulli L., Gabellone A., Legrottaglie A.R., Margari A., Margari L. (2021). Subjective and Electroencephalographic Sleep Parameters in Children and Adolescents with Autism Spectrum Disorder: A Systematic Review. J. Clin. Med..

[176] Cortesi F., Giannotti F., Ivanenko A., Johnson K. (2010). Sleep in Children with Autistic Spectrum Disorder. Sleep Med..

[177] Souders M.C., Zavodny S., Eriksen W., Sinko R., Connell J., Kerns C., Schaaf R., Pinto-Martin J. (2017). Sleep in Children with Autism Spectrum Disorder. Curr. Psychiatry Rep..

[178] Carmassi C., Palagini L., Caruso D., Masci I., Nobili L., Vita A., Dell’Osso L. (2019). Systematic Review of Sleep Disturbances and Circadian Sleep Desynchronization in Autism Spectrum Disorder: Toward an Integrative Model of a Self-Reinforcing Loop. Front. Psychiatry.

[179] Arazi A., Meiri G., Danan D., Michaelovski A., Flusser H., Menashe I., Tarasiuk A., Dinstein I. (2020). Reduced Sleep Pressure in Young Children with Autism. Sleep.

[180] Johnson K.P., Giannotti F., Cortesi F. (2009). Sleep Patterns in Autism Spectrum Disorders. Child Adolesc. Psychiatr. Clin..

[181] Schreck K.A., Mulick J.A., Smith A.F. (2004). Sleep Problems as Possible Predictors of Intensified Symptoms of Autism. Res. Dev. Disabil..

[182] Souders M.C., Mason T.B.A., Valladares O., Bucan M., Levy S.E., Mandell D.S., Weaver T.E., Pinto-Martin J. (2009). Sleep Behaviors and Sleep Quality in Children with Autism Spectrum Disorders. Sleep.

[183] DeVincent C.J., Gadow K.D., Delosh D., Geller L. (2007). Sleep Disturbance and Its Relation to DSM-IV Psychiatric Symptoms in Preschool-Age Children with Pervasive Developmental Disorder and Community Controls. J. Child Neurol..

[184] Liu X., Hubbard J.A., Fabes R.A., Adam J.B. (2006). Sleep Disturbances and Correlates of Children with Autism Spectrum Disorders. Child Psychiatry Hum. Dev..

[185] Leyfer O.T., Folstein S.E., Bacalman S., Davis N.O., Dinh E., Morgan J., Tager-Flusberg H., Lainhart J.E. (2006). Comorbid Psychiatric Disorders in Children with Autism: Interview Development and Rates of Disorders. J. Autism Dev. Disord..

[186] Yamada T., Watanabe T., Sasaki Y. (2023). Are Sleep Disturbances a Cause or Consequence of Autism Spectrum Disorder?. Psychiatry Clin. Neurosci..

[187] Veatch O.J., Maxwell-Horn A.C., Malow B.A. (2015). Sleep in Autism Spectrum Disorders. Curr. Sleep Med. Rep..

[188] Winkelman J.W., Buxton O.M., Jensen J.E., Benson K.L., O’Connor S.P., Wang W., Renshaw P.F. (2008). Reduced Brain GABA in Primary Insomnia: Preliminary Data from 4T Proton Magnetic Resonance Spectroscopy (1H-MRS). Sleep.

[189] Bridi M.C.D., Zong F.-J., Min X., Luo N., Tran T., Qiu J., Severin D., Zhang X.-T., Wang G., Zhu Z.-J. (2020). Daily Oscillation of the Excitation-Inhibition Balance in Visual Cortical Circuits. Neuron.

[190] Tamaki M., Watanabe T., Sasaki Y. (2021). Coregistration of Magnetic Resonance Spectroscopy and Polysomnography for Sleep Analysis in Human Subjects. STAR Protoc..

[191] Miano S., Bruni O., Elia M., Trovato A., Smerieri A., Verrillo E., Roccella M., Terzano M.G., Ferri R. (2007). Sleep in Children with Autistic Spectrum Disorder: A Questionnaire and Polysomnographic Study. Sleep Med..

[192] Diomedi M., Curatolo P., Scalise A., Placidi F., Caretto F., Gigli G.L. (1999). Sleep Abnormalities in Mentally Retarded Autistic Subjects: Down’s Syndrome with Mental Retardation and Normal Subjects. Brain Dev..

[193] Elia M., Ferri R., Musumeci S.A., Del Gracco S., Bottitta M., Scuderi C., Miano G., Panerai S., Bertrand T., Grubar J.C. (2000). Sleep in Subjects with Autistic Disorder: A Neurophysiological and Psychological Study. Brain Dev..

[194] Thirumalai S.S., Shubin R.A., Robinson R. (2002). Rapid Eye Movement Sleep Behavior Disorder in Children with Autism. J. Child Neurol..

[195] Limoges E., Mottron L., Bolduc C., Berthiaume C., Godbout R. (2005). Atypical Sleep Architecture and the Autism Phenotype. Brain.

[196] Xue M., Brimacombe M., Chaaban J., Zimmerman-Bier B., Wagner G.C. (2008). Autism Spectrum Disorders: Concurrent Clinical Disorders. J. Child Neurol..

[197] Ingiosi A.M., Schoch H., Wintler T., Singletary K.G., Righelli D., Roser L.G., Medina E., Risso D., Frank M.G., Peixoto L. (2019). Shank3 Modulates Sleep and Expression of Circadian Transcription Factors. Elife.

[198] Medina E., Schoch H., Ford K., Wintler T., Singletary K.G., Peixoto L. (2022). Shank3 Influences Mammalian Sleep Development. J. Neurosci. Res..

[199] Cochoy D.M., Kolevzon A., Kajiwara Y., Schoen M., Pascual-Lucas M., Lurie S., Buxbaum J.D., Boeckers T.M., Schmeisser M.J. (2015). Phenotypic and Functional Analysis of SHANK3 Stop Mutations Identified in Individuals with ASD and/or ID. Mol. Autism.

[200] MacDuffie K.E., Munson J., Greenson J., Ward T.M., Rogers S.J., Dawson G., Estes A. (2020). Sleep Problems and Trajectories of Restricted and Repetitive Behaviors in Children with Neurodevelopmental Disabilities. J. Autism Dev. Disord..

[201] MacDuffie K.E., Shen M.D., Dager S.R., Styner M.A., Kim S.H., Paterson S., Pandey J., St John T., Elison J.T., Wolff J.J. (2020). Sleep Onset Problems and Subcortical Development in Infants Later Diagnosed with Autism Spectrum Disorder. Am. J. Psychiatry.

[202] CDC CDC Child Development Positive Parenting Tips. https://www.cdc.gov/ncbddd/childdevelopment/positiveparenting/index.html.

[203] Hughes J.R. (2007). Autism: The First Firm Finding = Underconnectivity?. Epilepsy Behav..

[204] Supekar K., Uddin L.Q., Khouzam A., Phillips J., Gaillard W.D., Kenworthy L.E., Yerys B.E., Vaidya C.J., Menon V. (2013). Brain Hyperconnectivity in Children with Autism and Its Links to Social Deficits. Cell Rep..

[205] Noonan S.K., Haist F., Müller R.-A. (2009). Aberrant Functional Connectivity in Autism: Evidence from Low-Frequency BOLD Signal Fluctuations. Brain Res..

[206] O’Reilly C., Lewis J.D., Elsabbagh M. (2017). Is Functional Brain Connectivity Atypical in Autism? A Systematic Review of EEG and MEG Studies. PLoS ONE.

[207] Uddin L.Q., Supekar K., Menon V. (2013). Reconceptualizing Functional Brain Connectivity in Autism from a Developmental Perspective. Front. Hum. Neurosci..

[208] Haghighat H., Mirzarezaee M., Araabi B.N., Khadem A. (2021). Functional Networks Abnormalities in Autism Spectrum Disorder: Age-Related Hypo and Hyper Connectivity. Brain Topogr..

[209] Rane P., Cochran D., Hodge S.M., Haselgrove C., Kennedy D.N., Frazier J.A. (2015). Connectivity in Autism: A Review of MRI Connectivity Studies. Harv. Rev. Psychiatry.

